# Involvement of ILC1-like innate lymphocytes in human autoimmunity, lessons from alopecia areata

**DOI:** 10.7554/eLife.80768

**Published:** 2023-03-17

**Authors:** Rimma Laufer Britva, Aviad Keren, Marta Bertolini, Yehuda Ullmann, Ralf Paus, Amos Gilhar

**Affiliations:** 1 Skin Research Laboratory, Rappaport Faculty of Medicine, Technion – Israel Institute of Technology Haifa Israel; 2 Department of Dermatology, Rambam Health Care Campus Haifa Israel; 3 Monasterium Laboratory Münster Germany; 4 Department of Plastic Surgery, Rambam Medical Center Haifa Israel; 5 Dr. Phillip Frost Department of Dermatology & Cutaneous Surgery, Miller School of Medicine, University of Miami Miami United States; 6 CUTANEON Hamburg Germany; Yale University United States; The Feinstein Institute for Medical Research United States

**Keywords:** SCID/beige mice, xenotransplants, innate immunity, Mouse

## Abstract

Here, we have explored the involvement of innate lymphoid cells-type 1 (ILC1) in the pathogenesis of alopecia areata (AA), because we found them to be significantly increased around lesional and non-lesional HFs of AA patients. To further explore these unexpected findings, we first co-cultured autologous circulating ILC1-like cells (ILC1lc) with healthy, but stressed, organ-cultured human scalp hair follicles (HFs). ILClc induced all hallmarks of AA ex vivo: they significantly promoted premature, apoptosis-driven HF regression (catagen), HF cytotoxicity/dystrophy, and most important for AA pathogenesis, the collapse of the HFs physiological immune privilege. NKG2D-blocking or IFNγ-neutralizing antibodies antagonized this. In vivo, intradermal injection of autologous activated, NKG2D+/IFNγ-secreting ILC1lc into healthy human scalp skin xenotransplanted onto SCID/beige mice sufficed to rapidly induce characteristic AA lesions. This provides the first evidence that ILC1lc, which are positive for the ILC1 phenotype and negative for the classical NK markers, suffice to induce AA in previously healthy human HFs ex vivo and in vivo, and further questions the conventional wisdom that AA is always an autoantigen-dependent, CD8 +T cell-driven autoimmune disease.

## Introduction

Alopecia areata (AA) is both the most common inflammatory hair loss disorder and one of the most common human autoimmune diseases and exerts a major negative impact on quality of life (Gilhar et al., 2012; Gilhar et al., 2019a; Korta et al., 2018; Pratt et al., 2017). Despite major recent advances in AA therapy, a causal therapy does not yet exist, and disease relapse after therapy discontinuation is the rule, not the exception in long-standing AA (Meah et al., 2020; Gilhar et al., 2019a). Thus, the currently available, purely symptomatic AA therapy, including JAK inhibitors (Gilhar et al., 2019b), remains unsatisfactory. Since the exact pathobiology of AA and its clinical variants remains to be fully characterized, the – likely diverse – disease-initiating factors that ultimately result in the characteristic AA hair loss pattern shared by all AA variants, require more comprehensive dissection for optimal, personalized therapeutic targeting (Bertolini et al., 2020; Paus et al., 2018).

Specifically, there is increasing awareness that a classical, autoantigen- and CD8 +T cell-dependent autoimmune variant of AA (AAA) and a possibly autoantigen-independent non-autoimmune variant (NAIAA) may have to be distinguished from each other (Gilhar et al., 2019a; Bertolini et al., 2020; Paus et al., 2018; Paus, 2020). This is in line with the long-standing, but often under-appreciated clinical recognition that AA shows a wide spectrum of phenotypes and sub-forms (Gilhar et al., 2012; Ikeda, 1965; Meah et al., 2021; King et al., 2022).

One reason why the currently available AA therapy is not entirely satisfactory may be related to as yet insufficient therapeutic targeting of innate immunocytes in the immunopathogenesis of human AA, namely in NAIAA, even though these are now recognized as major players in AA pathobiology (Ghraieb et al., 2018; Ito et al., 2008; Li et al., 2016; Uchida et al., 2020; Uchida et al., 2021).

Previously, we had demonstrated that AA lesions are associated with a massive increase in the number of perifollicular NKG2D+NK cells (Gilhar et al., 2013a), which recognize the activating NKG2D ligand MICA, a ‘danger’ signal that is greatly overexpressed by the epithelium of lesional AA HFs (Ito et al., 2008; Li et al., 2016; Connell and Jabbari, 2022). Subsequent work has confirmed the key role of NKG2D and its activating ligands in human and murine AA (Xing et al., 2014; Petukhova et al., 2010). In fact, AA lesions can be induced experimentally in healthy human scalp skin in vivo by the transfer of interleukin 2 (IL-2)-activated NKG2D+ cells (Gilhar et al., 2013a), most of which had NK cell characteristics, with only a small minority of CD8 +T cells being present, that is the best-recognized pathogenic lymphocyte population in AA (Gilhar et al., 2012; Gilhar et al., 2013a; Pratt et al., 2017; Bertolini et al., 2020; de Jong et al., 2018). Moreover, pro-inflammatory mast cells (Bertolini et al., 2014) and (likely autoantigen-non-specific) γδ T-cells are also increased around/in lesional human AA HFs (Uchida et al., 2020). Finally, these ‘intermediate immunity’ protagonists suffice to induce the hallmarks of AA ex vivo (Uchida et al., 2021).

Taken together, this questions whether pathogenic, autoreactive CD8 +T cells are the only drivers of disease, and that all cases of AA, represent a genuine, autoantigen-dependent autoimmune disease (Bertolini et al., 2020; Paus et al., 2018) in the strictly defined sense of this term (Rose and Bona, 1993).

In our ongoing exploration of the role of innate/transitional immunity in the pathobiology of AA (Paus, 2020; Uchida et al., 2020; Uchida et al., 2021; Gilhar et al., 2019a; Bertolini et al., 2014), we, therefore, have asked in the current study whether innate lymphoid cells type 1 (ILC1 cells) (Zhou et al., 2020; Nabekura and Shibuya, 2021a; Colonna, 2018) can initiate human AA lesions.

We were interested in these immunocytes since human ILC1 cells secrete large amounts of interferon-γ (IFN-γ) (Ebbo et al., 2017), the crucial AA pathogenesis-promoting cytokine (Gilhar et al., 2012; Gilhar et al., 2019a; Paus et al., 2018), and this notably independent of classical autoantigen-specific CD8 +T cell activities. These ‘unconventional’ T-cells are placed in strategic tissue locations (Collins et al., 2017; Jiao et al., 2016; Kim et al., 2021) and represent an important link between innate and adaptive immunity (Vivier et al., 2018). While ILC1s play an essential role in human inflammatory bowel disease (IBD) (Ebbo et al., 2017; Luo et al., 2022; Clottu et al., 2021), their role in the pathophysiology of autoimmune hepatitis and rheumatoid arthritis requires further investigation (Ebbo et al., 2017; Fang et al., 2020; Yang et al., 2015), and their role in human autoimmune diseases overall remains insufficiently understood. We hypothesized that AA might offer a good model disease for interrogating this role.

ILC1 cells are classified as a component of type 1 immunity (Shannon et al., 2021), express NKG2D, recognize conserved phosphoantigens (Nabekura and Shibuya, 2021a), and contribute to immunity against tumor cells, for example through NKG2D activation (Dadi et al., 2016). The activating receptor NKG2D and its ligands (MICA, ULBP3) play an important role in innate (NK, ILC1), ‘translational’ (γδ T-cells) and CD8 T-cell-mediated immune responses to tumors and in several autoimmune diseases (Frazao et al., 2019; Babic and Romagnani, 2018).

Given that ILC1 cells produce TH1-type cytokines (such as IFN-γ) and share several phenotypic markers with NK cells, namely NKG2D (Spits et al., 2016), it is challenging to distinguish NKs and ILC1 cells (Tulic et al., 2019; Zhang et al., 2018; Seillet et al., 2021; Conlon et al., 2021). In fact, how to reliably discriminate between NK cells and ILC1s and unraveling the shared and distinct functions of these cell populations remains an important open quest (Seillet et al., 2021; Lopes et al., 2023; Cheng et al., 2023; Taggenbrock and van Gisbergen, 2023). For example, Eomes^hi^ T-bet^lo^ liver-resident NK cells have been described in humans and mice (Park et al., 2019; Harmon et al., 2016), while ILC1s from human tonsil and blood was also found to be Eomes+ (Cella et al., 2019). Therefore, the distinction between NK cells and ILC1s remains provisional – which is exactly why we have cautiously labeled the latter as ‘ILC1lc.’. The transcriptional and functional identity of ILC1 cells in humans is still a matter of debate, given that in contrast to other ILC subsets ILC1 cells seem to lack robust markers that enable their unequivocal identification and isolation (Bennstein et al., 2020).

However, although integrin α1 (CD49a) is upregulated on activated NK cells (Albini et al., 2021; Zheng et al., 2016), CD49a and integrin α2 (CD49b) are used as two mutually exclusive markers for distinguishing between NK and ILC1 cells, with NK cells being defined as CD49b+CD49a- and ILC1 as CD49b-CD49a+ (Gao et al., 2017; Vienne et al., 2021; Flommersfeld et al., 2021; Krzywinska et al., 2022) In the current study, we have accepted and employed this consensus. Also, in contrast to ILC1 and ILC1lc, classical NK cells demonstrate high T-bet and Eomes expression (T-bet^hi^ /Eomes^hi^) (Verma et al., 2020). Therefore, for the purpose of this study, we define ILC1lc as CD49a+CD49b- (Verma et al., 2020) and as lin-/CD127+/CD117-/CRTH2-phenotype, which are typical to classical ILC1 cells (Bennstein et al., 2020; Krabbendam et al., 2021), and also as T-bet^lo^/ Eomes^hi^ (Bennstein et al., 2020) (in contrast to classical T-bet^hi^ /Eomes^lo^ ILC1 cells Verma et al., 2020).

Specifically, we have asked whether (a) their number is increased in lesional AA skin, (b) they can damage human HFs ex vivo in a manner that mimics the AA phenotype, and finally (c) whether ILC1lc alone suffice to induce AA in previously healthy human scalp skin in vivo. To address these questions, we first analyzed the abundance, distribution, and phenotype of ILC1lc in human AA skin lesions compared to healthy human control skin. We then co-cultured autologous ILC1lc with freshly organ-cultured scalp HFs from the same patient, that is under conditions where the epithelium of these HFs transiently undergo an acute stress response and overexpresses MICA (Uchida et al., 2021), to check whether these innate lymphocytes exert any HFs cytotoxicity and/or impact on the physiological immune privilege (IP) of HFs (Bertolini et al., 2020; Paus et al., 2005; Ito et al., 2004; Peters et al., 2007; Bertolini et al., 2016). Finally, we injected autologous ILC1lc intradermally into healthy human scalp skin xenotransplants from the same human volunteers on SCID/beige mice to probe whether this suffices to induce classical AA hair loss lesions in vivo.

Taken together, our data show that ILC1lc is increased in AA lesions and suffice to induce an AA phenotype in healthy human HFs ex vivo and in vivo. This provides the first functional evidence of a key role of ILC1lc innate lymphocytes in a model human autoimmune disease (Colonna, 2018; Seillet et al., 2021; Conlon et al., 2021; Flommersfeld et al., 2021; Daussy et al., 2014; Park et al., 2019) - but also questions whether AA always a classical autoimmune disease is and underscore the role of innate immune cells in AA pathobiology.

## Results

### Peri- and intrafollicular infiltrates of ILC1lc are seen in both lesional and non-lesional AA skin

**Figure 1.**
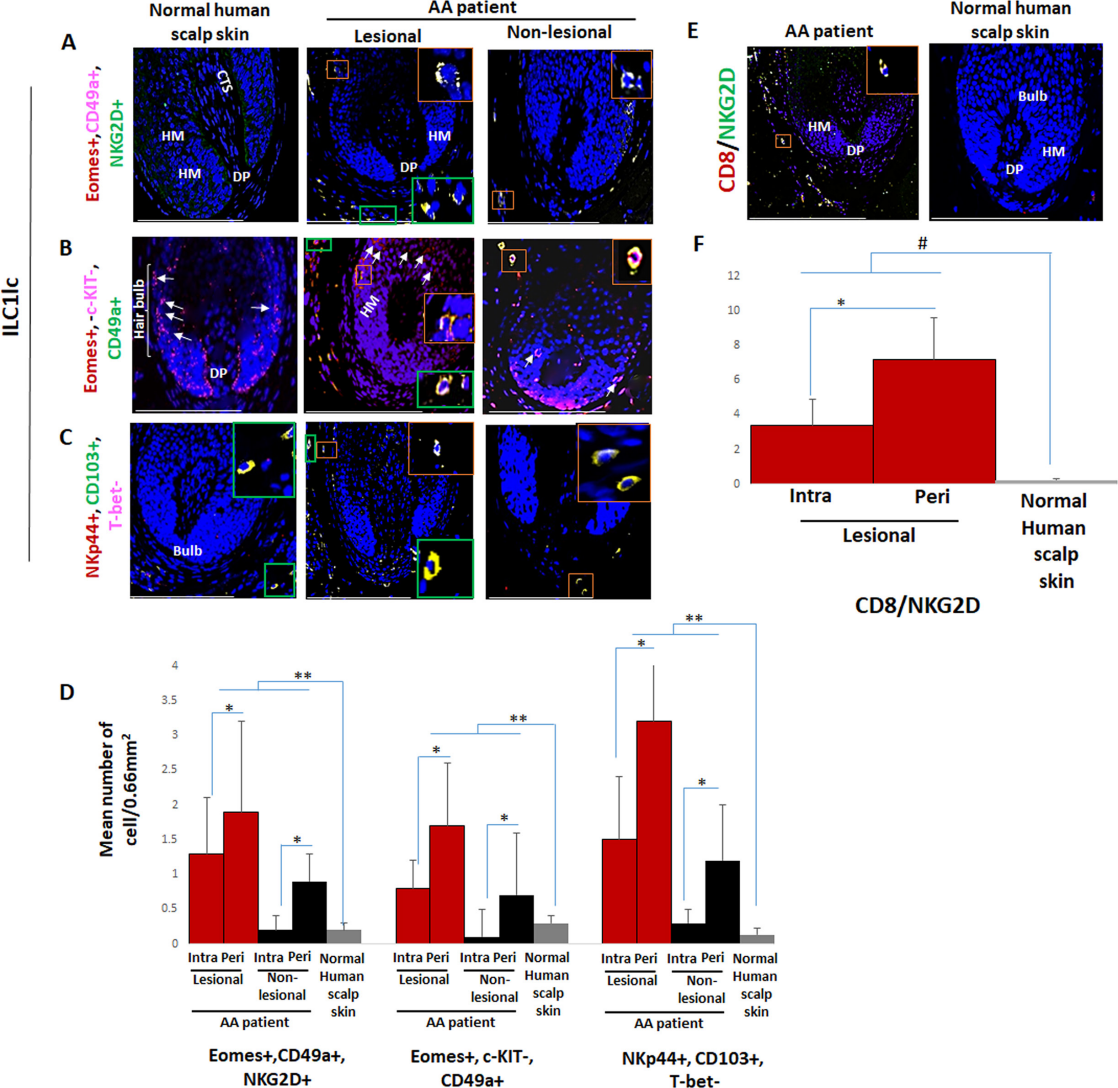
Immunofluorescence microscopy analyses of ILC1lc and CD8+/NKG2D+ cells in alopecia areata (AA) scalp skin. (**A**) ILC1lc (EOMES+, CD49a+, and NKG2D+) around HF in normal scalp skin, intrafollicular and perifollicular ILC1lc infiltrates in lesional and in non-lesional AA scalp patient. (**B**) EOMES+, c-KIT-,CD49a+, and (**C**) NKp44+, CD103+, T-bet- ILC1lc. For each panel, yellow staining indicates double staining A-EOMES+, NKG2D+; B- EOMES+, CD49a+; C- NKp44+,CD103+ (**D**) Quantitative immunohistomorphometry (qIHM) shows an increased number of ILC1lc in AA patients as compared to normal volunteers and increased number of the cells in lesional versus non-lesional areas of the patients. There is a significant increased perifollicular than intrafollicular ILC1lc in the lesional and non lesional areas. (**E**) CD8+/NKG2D+ cells around HF in AA scalp patient and absence of these cells in normal scalp skin of normal scalp skin. (**F**) There is an increased number of CD8+/NKG2D+ cells in HFs of AA patients compared to normal scalp skin and a significant lower number of ILC1lc versus CD8+/NKG2D+ cells in AA scalp skin. N=6 biopsies /AA patients and six biopsies /healthy donors from six independent donors, three areas were evaluated per section, and three sections per biopsy. Following Shapiro-Wilk test, Student’s *t*-test: *p<0.05, **p<0.01 or Mann Whitney *U* test: ^#^p<0.05. Scale bars, 50 µm. CTS- connective tissue sheath, DP - dermal papilla, HM - hair matrix, White arrow- c-KIT stained melanocyte. Figure 1—source data 1.Quantitative data for immunofluorescence microscopy analyses of ILC1lc and CD8+/NKG2D+ cells in AA scalp skin.

First, we investigated whether healthy and AA-affected human skin differs in their content and/or distribution of ILC1lc, using a comprehensive set of triple-immunofluorescence (IF) staining best suited to identify these immunocytes (Seillet et al., 2021; Bennstein et al., 2020; Gao et al., 2017). This revealed the presence of only extremely few ILC1lc in healthy control skin with all three staining settings employed (Eomes+, CD49a+, NKG2D+ [Figure 1A and Figure 1—figure supplement 1A], Eomes+, c-KIT-, CD49a+ [Figure 1B and Figure 1—figure supplement 1A], or NKp44+, CD103+, T-bet- cells [Figure 1C and D and Figure 1—figure supplement 1A; Kim, 2015; Fuchs et al., 2013; Salimi and Ogg, 2014]). These cells appeared to be preferentially scattered along the papillary dermis of healthy scalp skin biopsies and around the HFs (Figure 1C). This is reminiscent of the few Vδ1+T cells detectable in healthy human skin that also have a preferential perifollicular location and may ‘police’ the skin for molecular indications of tissue stress, namely of HFs (Uchida et al., 2020; Uchida et al., 2021).

**Figure 1—figure supplement 1.**
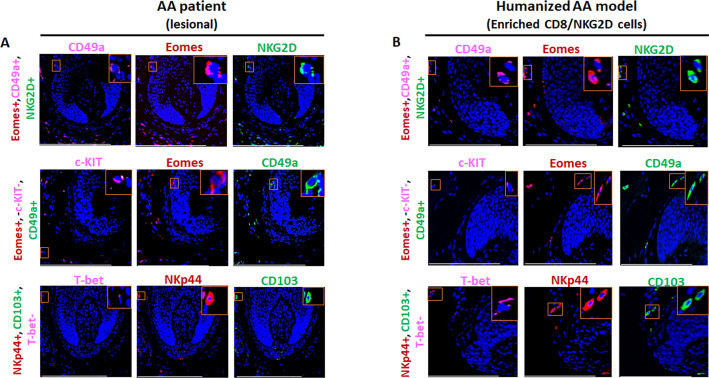
Single channels immunofluorescence microscopy analyses of various markers in AA scalp skin and in AA-induced xenotransplants. (**A**) Single channels of EOMES, T-bet, CD49a+, NKG2D+, c-kit, CD103, and NKp44 expressing cells in alopecia areata (AA) scalp patient and (**B**) in the humanized AA model. N=4 biopsies from AA patients and five biopsies from the humanized AA mouse model. Scale bars, 50 µm.

Instead, intra and peri-follicular infiltrates of ILC1lc were frequently present in lesional AA HFs (Figure 1A, B, C and D and Figure 1—figure supplement 1A), typically in conjunction with a dominant infiltrate of CD8+/NKG2D+ cells around the hair bulb (p<0.05) (Figure 1E and F). Importantly, the number of ILC1lc was already significantly increased in/around non-lesional AA HFs compared to healthy scalp skin (p<0.01) (Figure 1A, B, C and D and Figure 1—figure supplement 1A). This may indicate that ILC1lc may actually have arrived around the HFs before the CD8 cells and may have contributed to attracting the CD8 cells into the perifollicular space.

This strongly suggested that ILC1lc are not mere bystanders attracted only secondarily to the HFs by CD8 T-cells, similar to, but more pronounced than we have recently observed regarding perifollicular Vδ1+T cells in non-lesional AA skin (Uchida et al., 2020). This invited the hypothesis that ILC1lc is actively involved in transforming healthy human scalp HFs into lesional AA HFs.

### T-bet^lo^/Eomes^hi^ ILC1lc can be expanded from human peripheral blood mononuclear cells (PBMCs) in vitro

**Figure 2.**
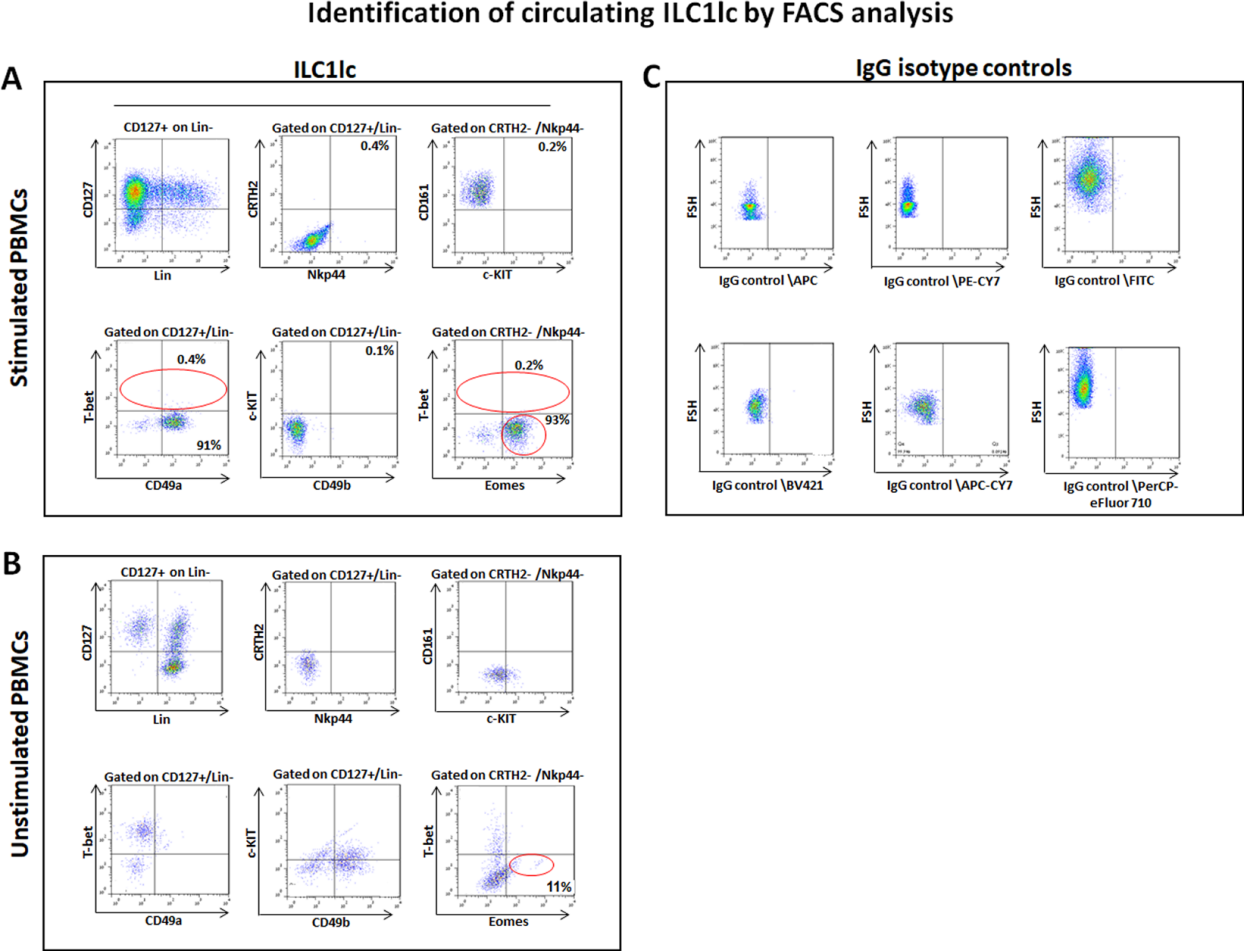
Circulating ILC1lc expanded and characterized by FACS analysis. (**A**) PBMCs activated by IL-18, IL-33 and IL-12 were sorted by FACS Aria and characterized by FACS analysis. ILC1lc markers were identified by the expression of CD127+, CD161+, c-KIT-, and CRTH2-, high levels of integrin α1 (CD49a) expression, combined with the absence of integrin α2 (CD49b) and transcription factors Eomes^hi^ and T-bet^lo^ (**B**) unstimulated PBMCs (**C**) isotype controls. N=10 blood donors, 1.5 × 10^6^ cells/blood donor, analysis was performed in triplicates from each of the blood donors. Following Shapiro-Wilk test, Student’s t-test, p<0.05. Figure 2—source data 1.Quantitative data for circulating ILC1lc expanded and characterized by FACS analysis.

To functionally probe this hypothesis, we isolated, purified, and characterized human peripheral blood-derived ILC1lc as the most suitable cell source for the planned HF-immunocyte co-culture studies. The scarcity of ILC1lc in healthy human skin, compared to their relative abundance in peripheral blood (Colonna, 2018; Artis and Spits, 2015) necessitated to isolate autologous ILC1lc from the latter source rather than from skin (Teunissen et al., 2014). To facilitate ILC1lc isolation, PBMCs of healthy volunteers were first cultured with high-dose IL-2 (100 U/mL) in the presence of IL-18 (1 µg/1 ml), IL-33 (1.5 µg/5 ml), and IL-12 (1.5 µg/5 ml), since these cytokines induce ILC1lc expansion (Salimi and Ogg, 2014; Silver et al., 2016; Orimo et al., 2020; Ohne et al., 2016). When ILC1lc were sorted by FACS Aria and characterized by FACS analysis on day seven of culture, low T-bet, and high Eomes expression were observed (Figure 2A), in contrast to classical T-bet^hi^ and Eomes^lo^ ILC1 cells (Jiao et al., 2016; Vivier et al., 2018; Zhang et al., 2018). In addition, the ILC1lc expressed and shared the following markers with classical ILC1 cells: LIN- CD3/CD1a/D14/CD19/CD34/CD123/CD11c /BDCH2/FcεR1α/TCRαβ/TCRγδ/CD56, CD127+, CD161+, c-KIT-, and CRTH2- (Zook and Kee, 2016; Bernink et al., 2017; Simoni and Newell, 2017; Figure 2A).

**Figure 2—figure supplement 1.**
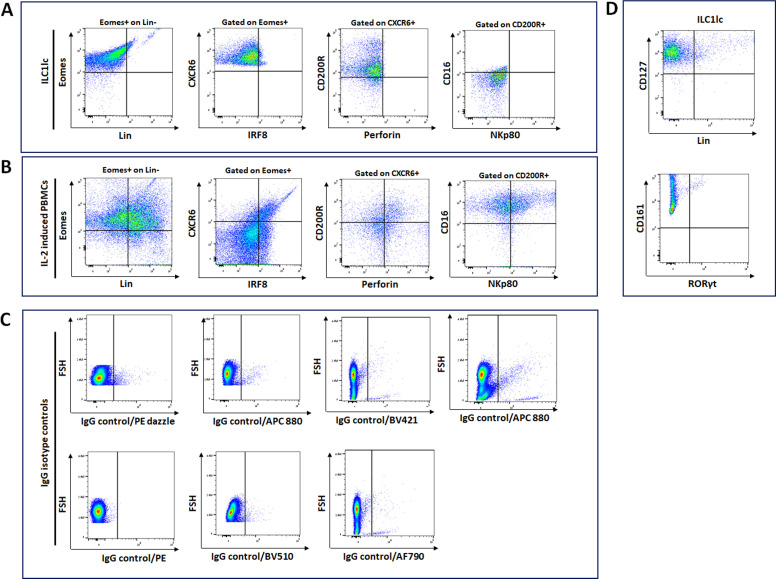
Circulating ILC1lc expanded and characterized by FACS analysis. (**A**) PBMCs activated by IL-18, IL-33, and IL-12 were sorted by FACS Aria and characterized by FACS analysis. ILC1-like cell markers were identified by the expression of Eomes+, CXCR6+, CD200R+, IRF8-, Perforin-, CD16-, and NKP80 (**B**) IL-2 induced PBMCs (**C**) isotype controls. (**D**) expression of CD127+, CD161 +, and RORγt-. N=4 blood donors, 1.5 × 10^6^ cells/blood donor, analysis was performed in triplicates from each of the blood donors. Following Shapiro-Wilk test, Student’s t-test, p<0.05.

**Figure 2—figure supplement 2.**
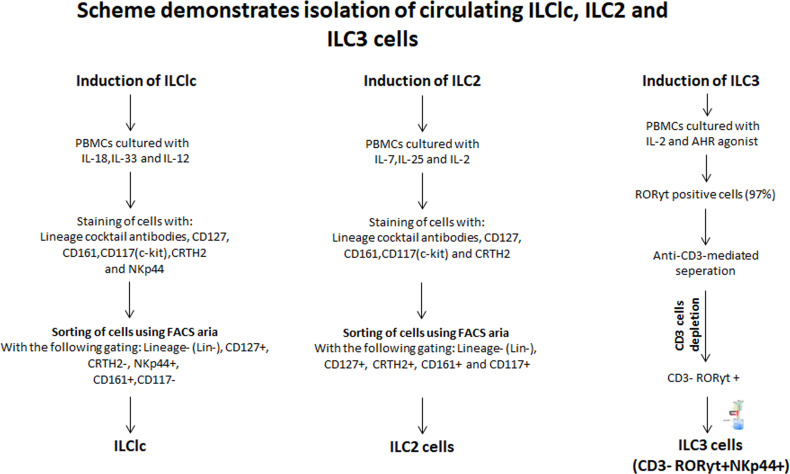
Scheme demonstrating the isolation of ILC1lc, ILC2s, and ILC3s cells from PBMCs of healthy human volunteers.

This immune phenotype suggests that the immune cells used in our study are best classified as ILC1lc (Nabekura and Shibuya, 2021a), and documents that all experiments reported below were indeed performed with autologous ILC1lc rather than with NK cell subpopulations. Indeed, the FACS analysis (Figure 2—figure supplement 1A,B and C) revealed that ILC1lc demonstrates the ILC1 phenotype (CD200R, CD127, CXCR6) (Lopes et al., 2023; Curio and Belz, 2022) but not of the classical NK cell lineage (IRF8, Perforin, NKp80, CD16) (Sagebiel et al., 2019; Brownlie et al., 2021; Krämer et al., 2023), thus further serving as an evidence that EOMES +ILCs represent distinct ILC1 lineage-defining markers. In contrast to NK cells, ILC1lc also expressed the expected high levels of integrin α1 (CD49a), combined with the absence of integrin α2 (CD49b) (Jiao et al., 2016; Figure 2A). All these characteristic markers of ILClc were absent in the control unstimulated PBMCs (Figure 2B and C).

This immune phenotype suggests that the immune cells used in our study are best classified as ILC1lc (Nabekura and Shibuya, 2021a), and documents that all experiments reported below were indeed performed with autologous ILC1lc rather than with NK cell subpopulations. Note that we had previously shown that NKG2D+/CD56 +NK cells suffice to induce AA lesions in human skin in vivo (Gilhar et al., 2013a; Laufer Britva et al., 2020) while iNKT cells are AA-protective in the humanized AA mouse model (Ghraieb et al., 2018). Subsequently, these ILC1lc were either used for HF co-culture assays or injected into healthy human scalp skin xenotransplants on SCID/beige mice (Gilhar et al., 2013a; Ito et al., 2005b). As controls, we also isolated ILC2 and ILC3 cells, which failed to induce AA phenotype in a sharp contrast to the ILC1lc (see Materials and methods).

In order to exclude the possibility that contamination from ILC3s during sorting the ILC1lc and thus contributing to the observed results, we generated a new set of FACS data on sorted ILC1lc. Given that ILC1 cells are RORγt negative while ILC3 cells are RORγt positive (Peng et al., 2022; Fiancette et al., 2021), the data clearly demonstrate that the contamination hypothesis is highly unlikely (Figure 2—figure supplement 1D).

### ILC1lc induces HF cytotoxicity ex vivo

Next, we functionally probed the interaction of ILC1lc with HFs that were investigated here as a model human (mini-) organ in which the interactions of a healthy human tissue system with defined, autologous immunocyte populations can be interrogated ex vivo in the absence of any confounding systemic immune or neural inputs (Uchida et al., 2021). For this, microdissected, organ-cultured human scalp HFs (Langan et al., 2015) were co-cultured for six days with autologous, peripheral blood-derived, purified, IL-12/IL-18/IL-33-prestimulated ILC1lc, or with autologous human CD8 +NKG2D+ cells (=positive control), ILC2, ILC3 cells, or PBMCs non-speciﬁcally activated with PHA (PBMCs/PHA) (=negative controls).

Importantly, only scalp HFs in the anagen VI stage of the hair cycle were used (identified as described) (Kloepper et al., 2010) that had been freshly placed into HF organ culture for 24 hr, since these HFs are maximally ‘stressed,’ in contrast to non-cultured HFs, that is immediately after isolation, or that had already undergone several days of adjusting to the harsh conditions of serum-free organ culture (Uchida et al., 2021; Langan et al., 2015). These ‘stressed’ day 1 HFs temporarily up-regulate MHC class Ia and ß2-microglobulin while the expression of IP guardians, that is αMSH and TGFβ2 remain unchanged (Uchida et al., 2021)*,* indicating a transiently weakened, but partially maintained HF immune privilege (Bertolini et al., 2020; Ito et al., 2004). The expression of molecules associated with tissue stress, that is the intrafollicular produced neurohormone, CRH (Ito et al., 2005a), and the NKG2D ligand MICA/B is also higher in day 1 organ-cultured HFs compared to freshly microdissected HFs or after day 3 of organ culture. Day 1 HFs also show signs of mild HF dystrophy (as evidenced by increased lactate dehydrogenase [LDH] release into the medium), and express chemokines recognized for their relevance in AA pathobiology, that is CXCL10 and CXCL12 (Uchida et al., 2021; Ito et al., 2020). Thus, day 1 HFs are ideally suited for interrogating human immunocyte interactions with a transiently ‘stressed,’ but otherwise healthy human (mini-) organ that overexpresses the NKG2D-activating ‘danger’ signal, MICA/B, under physiologically relevant ex vivo conditions (Uchida et al., 2021; Langan et al., 2015).

**Figure 3.**
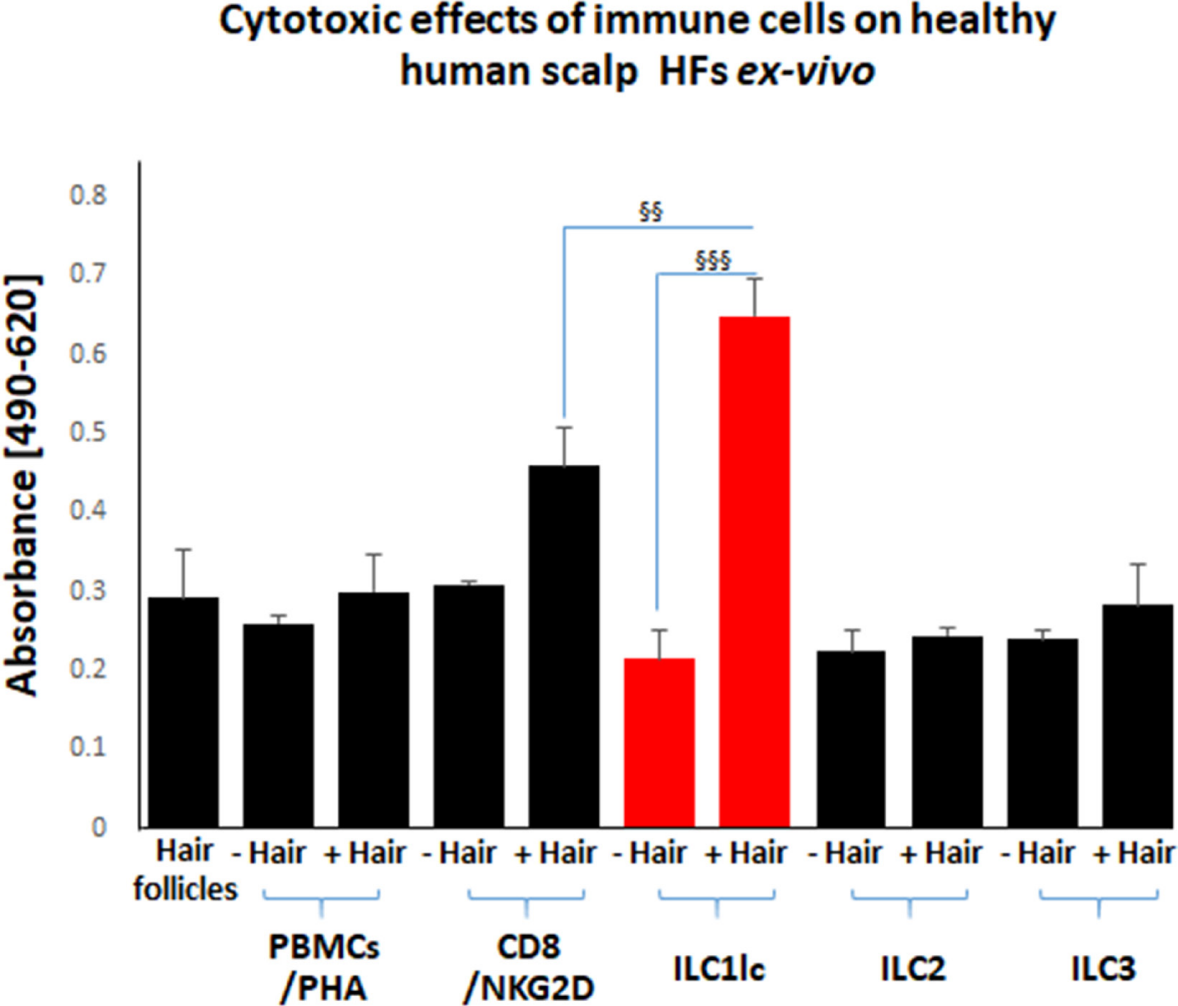
Cytotoxic effects of CD8+/NKG2D+and ILC1lc on normal human scalp HF ex vivo. These cell populations were placed separately into wells with (+Hair) dissected HFs and without (-Hair). Cytotoxic effects of these cell populations on normal human scalp HF ex vivo were studied by measuring the spontaneous release of lactate dehydrogenase (LDH) from the microdissected HFs. Increased cytotoxicity of ILC1lc co-cultured with HFs compared to CD8+/NKG2D+, as well as to ILC2s and ILC3s, and PBMCs/PHA cells. N=20–24 HFs/group derived from three independent donors analyzed in three independent HF organ culture experiments. Following Shapiro-Wilk test and Dunn’s test ^§^p<0.05, ^§§^p<0.01, ^§§§^p<0.001. Figure 3—source data 1.Quantitative data for cytotoxic effects of CD8+/NKG2D+ and ILC1lc on normal human scalp HF ex vivo*.*

**Figure 4.**
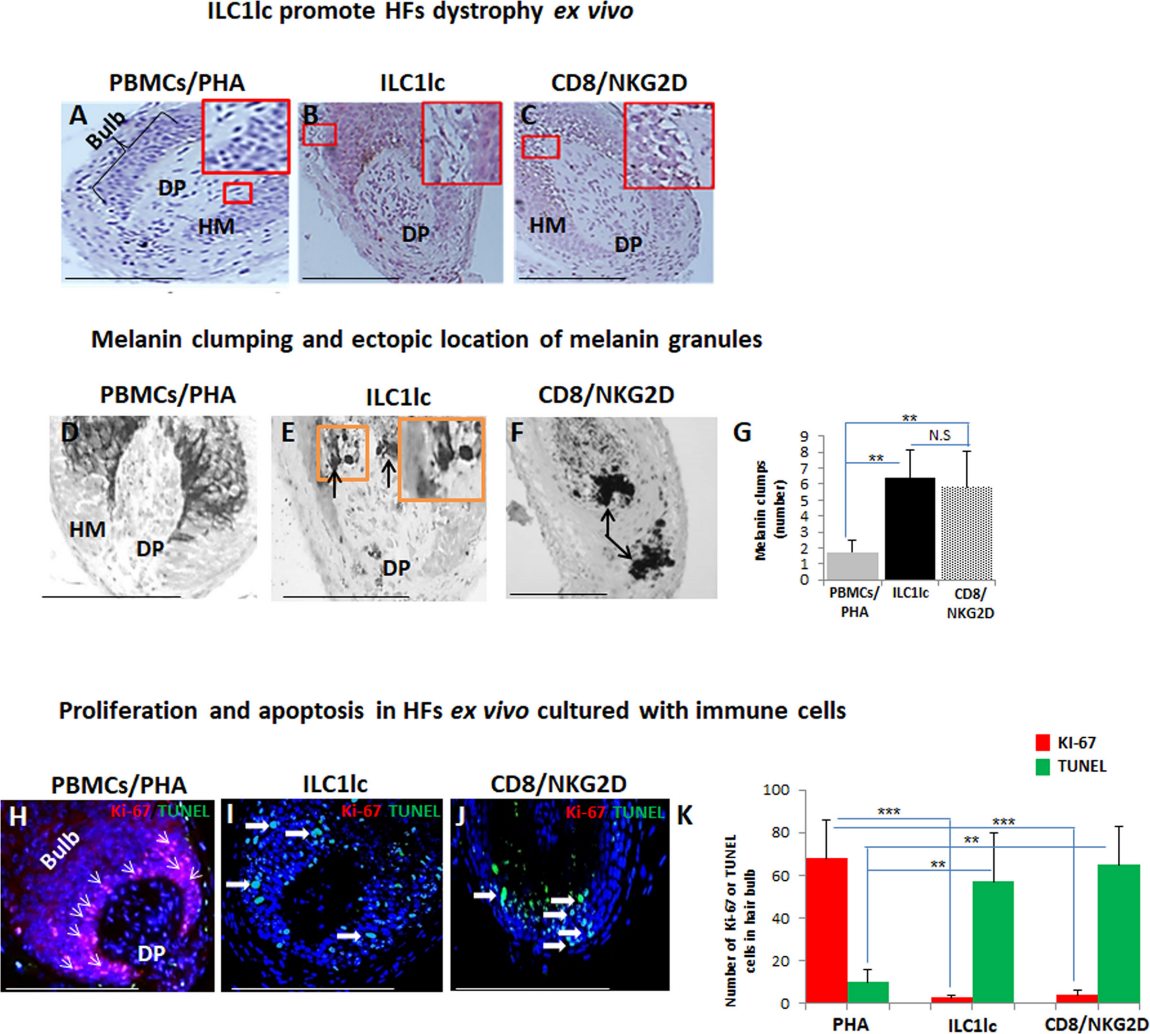
Hair follicles (HFs) dystrophy, melanin clumping, and apoptosis in normal human scalp HF ex vivo co-cultured with ILC1lc and CD8+/NKG2D+ cells. (**A–C**) H&E staining revealed undifferentiated and prominent matrix cells, condensed dermal papilla, and the appearance of apoptotic cells, N=15–19 HFs/group from three independent donors. (**D–G**) Masson-Fontana histochemistry revealed melanin clumping and ectopic location of melanin granules only in HFs co-cultured with CD8+/NKG2D+and ILC1lc, but not in HFs cultured with PBMCs/PHA. N=7–11, HFs/group from three independent donors. Following Shapiro-Wilk test,Student’s *t*-test: *p<0.05, **p<0.01, ***p<0.001. (**H–K**) HFs co-cultured with ILC1lc or CD8+/NKG2D+ cells showed a significantly decreased proliferation (pink, arrowhead) and increased apoptosis (green, wide arrows). N=6 HFs/group from two independent donors, three areas were evaluated per section. Following Shapiro-Wilk test, Student’s *t*-test: *p<0.05, **p<0.01, ***p<0.001 in the anagen hair bulb compared to HFs cultured with PBMCs/PHA. Scale bars, 50 µm. DP - dermal papilla, HM - hair matrix. Figure 4—source data 1.Quantitative data for HFs dystrophy, melanin clumping and apoptosis in normal human scalp HF ex vivo co-cultured with ILC1lc and CD8+/NKG2D+ cells.

First, we studied the cytotoxic effects of ILC1lc on healthy human scalp HF ex vivo by measuring the HF release of LDH into the culture medium. This not only showed significantly higher LDH release induced by ILC1lc than by co-culture with all three negative control cell populations (ILC2s, ILC3s, or PBMCs/PHA) but also even higher HF cytotoxicity levels than those induced by CD8+/NKG2D+ cells (p<0.01), namely after three days of co-culture (Figure 3). These HF cytotoxicity results were fully corroborated by characteristic morphological signs of HF dystrophy following co-culture with ILC1lc; while CD8+/NKG2D+ cells induced similar dystrophy phenomena, these were not seen after co-culture with PBMC/PHA (Figure 4A, B and C). The induction of significant HF dystrophy by ILC1lc ex vivo was further documented by the presence of pathological melanin clumping and ectopically located intrafollicular melanin granules (Bodó et al., 2007; Hendrix et al., 2005; Figure 4D, E, F and G) and by decreased proliferation and increased apoptosis of hair matrix keratinocytes (Figure 4H, I, J and K). Both were also seen in the CD8+/NKG2D+group (positive control), but not in HFs co-cultured with PBMCs/PHA (negative control) (p<0.001, p<0.01, respectively). Thus, autologous ILC1lc alone suffice to induce substantial HF cytotoxicity ex vivo if co-cultured with transiently ‘stressed,’ but otherwise healthy human scalp HFs.

### ILC1lc induces HF immune privilege collapse ex vivo via NKG2D stimulation

**Figure 5.**
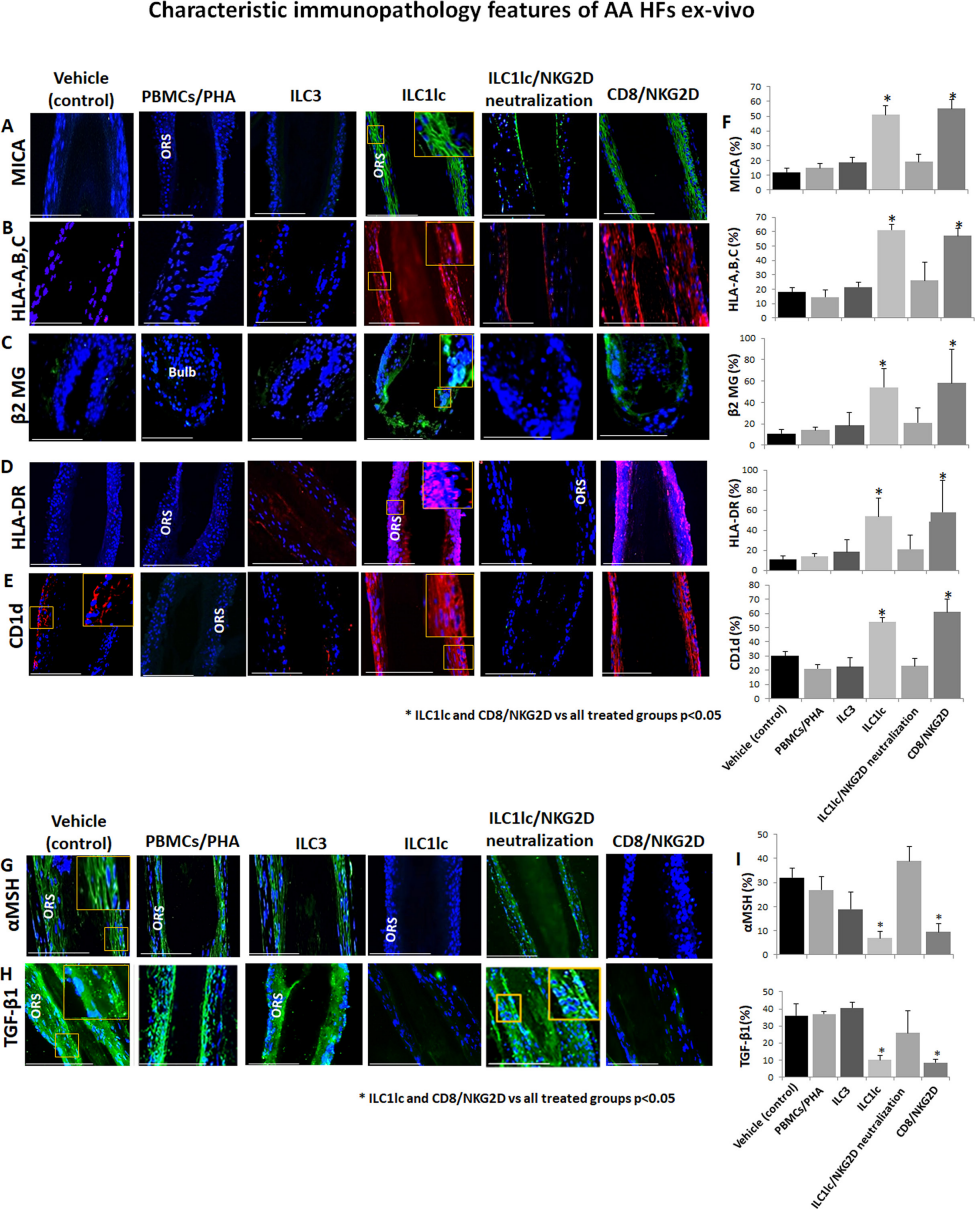
Characteristic immunopathology features of alopecia areata (AA) hair follicles (HFs). (**A**) MICA, (**B**) HLA-A,B,C, (**C**) β2 MG, (**D**) HLA-DR, and (**E**) CD1d, expression by HFs epithelium, which had been co-cultured with either ILC1lc or CD8+/NKG2D+ cells but not in the control HFs, which had been co-cultured with either ILC3s, PBMCs/PHA, ILC1lc /NKG2D neutralization or in the untreated HFs. (**F**) quantitation. (**G**) The immune inhibitory HF immune privilege guardians, α-MSH and (**H**) TGF-β1 almost disappeared in HFs/ ILC1lc and HFs/NKG2D but were prominently present in ILC1lc /NKG2D neutralization and control HFs, N=9–12 HFs/group from three independent donors, three areas were evaluated per section. Following Shapiro-Wilk test,Student’s t-test, *p<0.05. Scale bar, 100 µm. ORS - outer root sheet. Figure 5—source data 1.Quantitative data for characteristic immunopathology features of AA HFs.

Given that AA cannot occur without the prior collapse of HF immune privilege [HF-IP] (Gilhar et al., 2012; Bertolini et al., 2020), we also investigated the impact of ILC1lc on key HF-IP markers. Indeed, the co-culture of HFs with ILC1lc triggered IP collapse, as evidenced by ectopic and overexpressed HLA-A,B,C, ß2-microglobulin (ß2 MG), and HLA-DR, along with overexpression of the ‘danger’/tissue distress signals, MICA and CD1d, which interact with and stimulate NKG2D (Uchida et al., 2021; Fan et al., 2022) as compared to HFs interacting with PBMC/PHA or with ILC3 cells (Figure 5A, B, C, D, E and F).

Notably, quantitative immunohistomorphoemtry (qIHM) also showed that protein expression of the immunoinhibitory HF-IP guardians, TGF-β1 and α-MSH (Gilhar et al., 2012; Bertolini et al., 2020; Paus et al., 2018; Ito et al., 2004), almost disappeared in the epithelium of HFs co-cultured with autologous ILC1lc or CD8+/NKG2D+ cells (=positive control) (Figure 5G, H and I)**,** while these critical HF-IP guardians were still prominently expressed in negative control HFs (Figure 5G, H and I). Importantly, adding anti-NKG2D blocking antibodies prevented HFs IP collapse and preserved the IP in the ILC1lc /NKG2D treated group (Figure 5G, H and I).

This demonstrates that autologous ILC1lc induces human HF-IP collapse ex vivo – incidentally, the first time that the induction of IP collapse by ILC1lc has been documented in an intact human tissue/organ.

### ILC1lc are activated by ‘stressed’ HFs and induce premature catagen development via IFN-γ secretion

**Figure 6.**
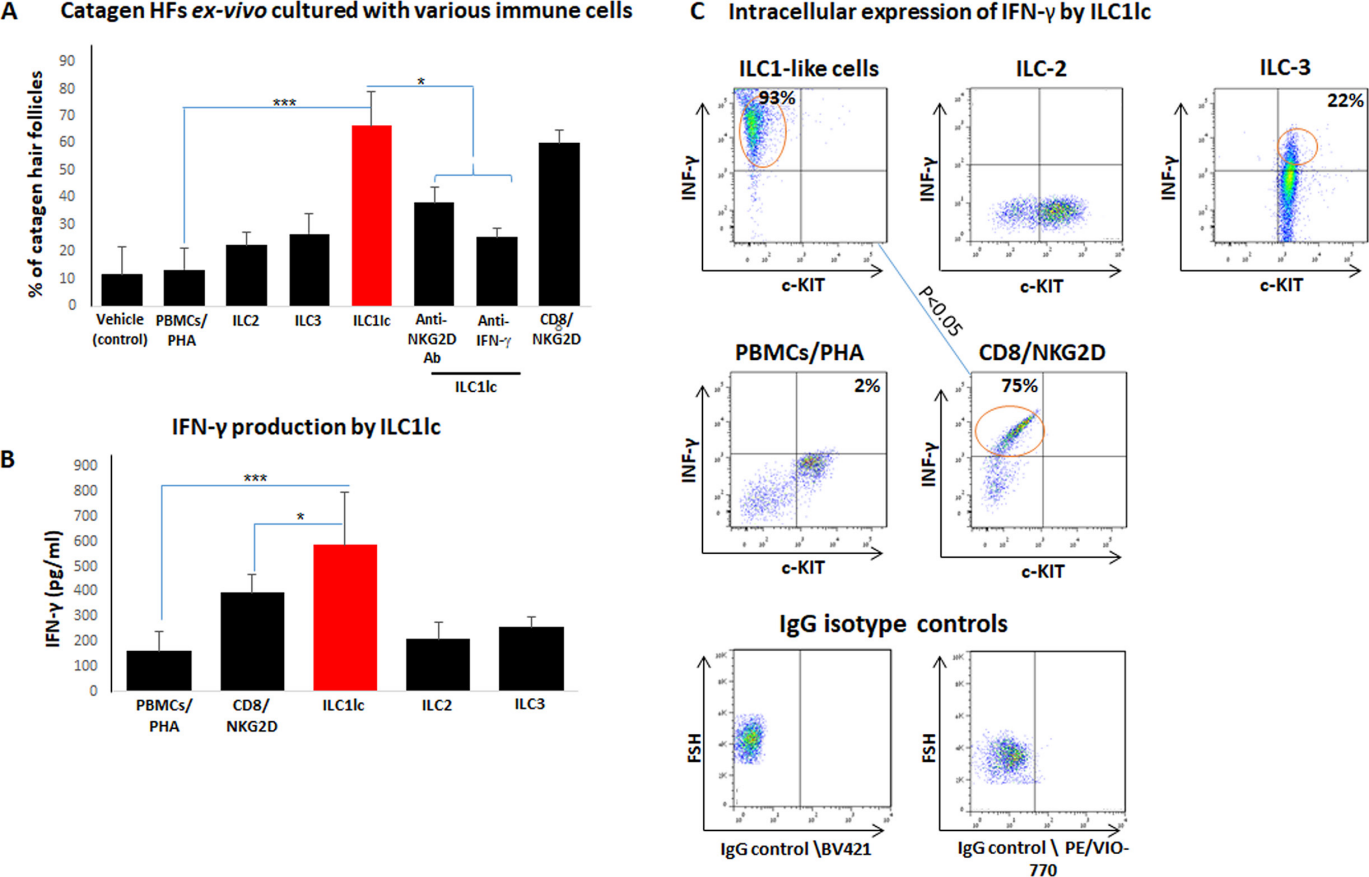
Transition of anagen to catagen hair follicles (HFs) following culture with ILC1lc or CD8+/NKG2D+ cells in human scalp HF ex vivo. (**A**) These immune cells significantly accelerated the transformation of anagen HFs into catagen HFs ex vivo compared to ILC2, ILC3, PBMCs/PHA, and neutralizing anti- IFN-γ, anti-NKG2D antibodies. N=28–34 HFs/group taken from six independent donors, Student’s *t*-test: *p<0.05, **p<0.01, ***p<0.001. (**B**) ELISA analysis revealed increased IFN-γproduction by ILC1lc /HFs compared to production by CD8+/NKG2D+ cells, ILC2s, ILC3s, and PBMCs/PHA. N=6 healthy donors, 6 × 10^6^ cells from each donor. Following Shapiro-Wilk test, Student’s t-test: *p<0.05, **p<0.01, ***p<0.001. (**C**) FACS analysis revealed a significant increased intracellular IFN-γexpression in ILC1lc co-cultured with HFs compared to the effector CD8+/NKG2D+and to ILC2s and ILC3s, N=6 blood donors, 1.5 × 10^6^ cells/blood donor. Student’s *t*-test, p<0.05. Figure 6—source data 1.Quantitative data for transition of anagen to catagen HFs following culture with ILC1lc or CD8+/NKG2D+ cells in human scalp HF ex vivo.

Next, we examined how autologous ILC1lc impacted on human HF cycling, given that premature induction of apoptosis-driven HF regression (catagen) is one of the hallmarks of AA (Gilhar et al., 2012; Bertolini et al., 2020; Messenger et al., 1986). This showed that ILC1lc significantly accelerated the transformation of anagen into catagen HFs ex vivo (Paus et al., 2005) compared to all three negative controls (ILC2, ILC3, or PBMCs/PHA) – thus eliciting the third hallmark of the AA phenotype besides HF-IP collapse and dystrophy ex vivo (Gilhar et al., 2012; Bertolini et al., 2020; Messenger et al., 1986; Figure 6A), just as we had previously shown for Vδ1+ γδT cells (Uchida et al., 2021). As expected (Gilhar et al., 2012; Pratt et al., 2017; Bertolini et al., 2020; de Jong et al., 2018; Xing et al., 2014), premature catagen induction was also seen with CD8+/NKG2D+ cells (=positive control), but not with any of the negative control cell populations (Figure 6A).

ILC1lc prominently secrete IFN-γ (Seillet et al., 2021), that is the cytokine that we had shown to induce HF damage (dystrophy), premature catagen, and HF-IP collapse most potently (Ito et al., 2004; Ito et al., 2005a). Therefore, we next investigated IFN-γrelease in these co-culture experiments. ELISA analysis revealed that ILC1lc produced and secreted higher amounts of IFN-γ into the medium than all other cells co-cultured with ‘stressed’ HFs, including CD8+/NKG2D+ cells (p<0.05) (Figure 6B). This suggests that ILC1lc possesses even stronger HF cytotoxicity-, IP collapse- and dystrophy-inducing properties than CD8 +T cells, the classical effector cells of AAA (Gilhar et al., 2012; Pratt et al., 2017; Bertolini et al., 2020).

FACS analysis showed ILC1lc activation when these were co-cultured with ‘stressed,’ autologous HFs (600 cells/HF), as evidenced by significantly increased intracellular IFN-γ expression by ILC1lc (Bernink et al., 2017) (93 ± 11%) compared to the positive control (CD8+/NKG2D+, 75 ± 9%, p<0.05) (Figure 6C) and the negative controls ILC2s, 11 ± 1%, p<0.05; ILC3s, 28 ± 5%, p<0.05; PBMCs/PHA, 2 ± 1.3%, p<0.001 (Figure 6C).

When neutralizing anti- IFN-γ antibodies were administered into the medium of the organ culture, premature catagen development of HFs co-cultured with ILC1lc was significantly reduced (Figure 6A), strongly suggesting that premature catagen induction by ILC1lc depends on their IFN-γsecretion (Seillet et al., 2021). Importantly, reduced catagen induction was also seen after adding NKG2D-blocking antibodies to the medium (Figure 6A). This suggests that ILC1lc activation and IFN-γsecretion are induced by NKG2D-stimulating danger signals overexpressed by stressed HF epithelium, such as MICA. These findings further support the recognized central role of both IFN-γand NKG2D in the initial stages of AA pathobiology (Gilhar et al., 2012; Paus et al., 2018; Ito et al., 2008; de Jong et al., 2018).

### ILC1lc suffice to induce AA lesions in healthy human scalp skin in vivo

Taken together, these clinically relevant ex vivo experiments documented that ILC1lc can indeed induce the hallmarks of AA in healthy human scalp HFs ex vivo: HF-IP collapse, HF dystrophy, and premature catagen development (Gilhar et al., 2012; Paus et al., 2018). Therefore, we finally probed the hypothesis that ILC1lc may also suffice to induce human AA-like hair loss lesions in vivo using our established humanized AA mouse model (Gilhar et al., 2013a; Ghraieb et al., 2018; Gilhar et al., 2016). We had previously demonstrated that a macroscopic and histological phenocopy of human AA lesions can be rapidly induced experimentally in healthy human scalp skin xenotransplants on SCID/beige mice in vivo by the intradermal injection of enriched CD8/NKG2D are defined as PBMCs that have been cultured for 14 days in high-dose IL-2 (100 U/ml) according to our previously published characterization (Ghraieb et al., 2018; Gilhar et al., 2013a; Gilhar et al., 2013b; Gilhar et al., 2016; Bertolini et al., 2014). These cells are derived from healthy donors, that is in the absence of a specific genetic or autoimmune constellation.

**Figure 7.**
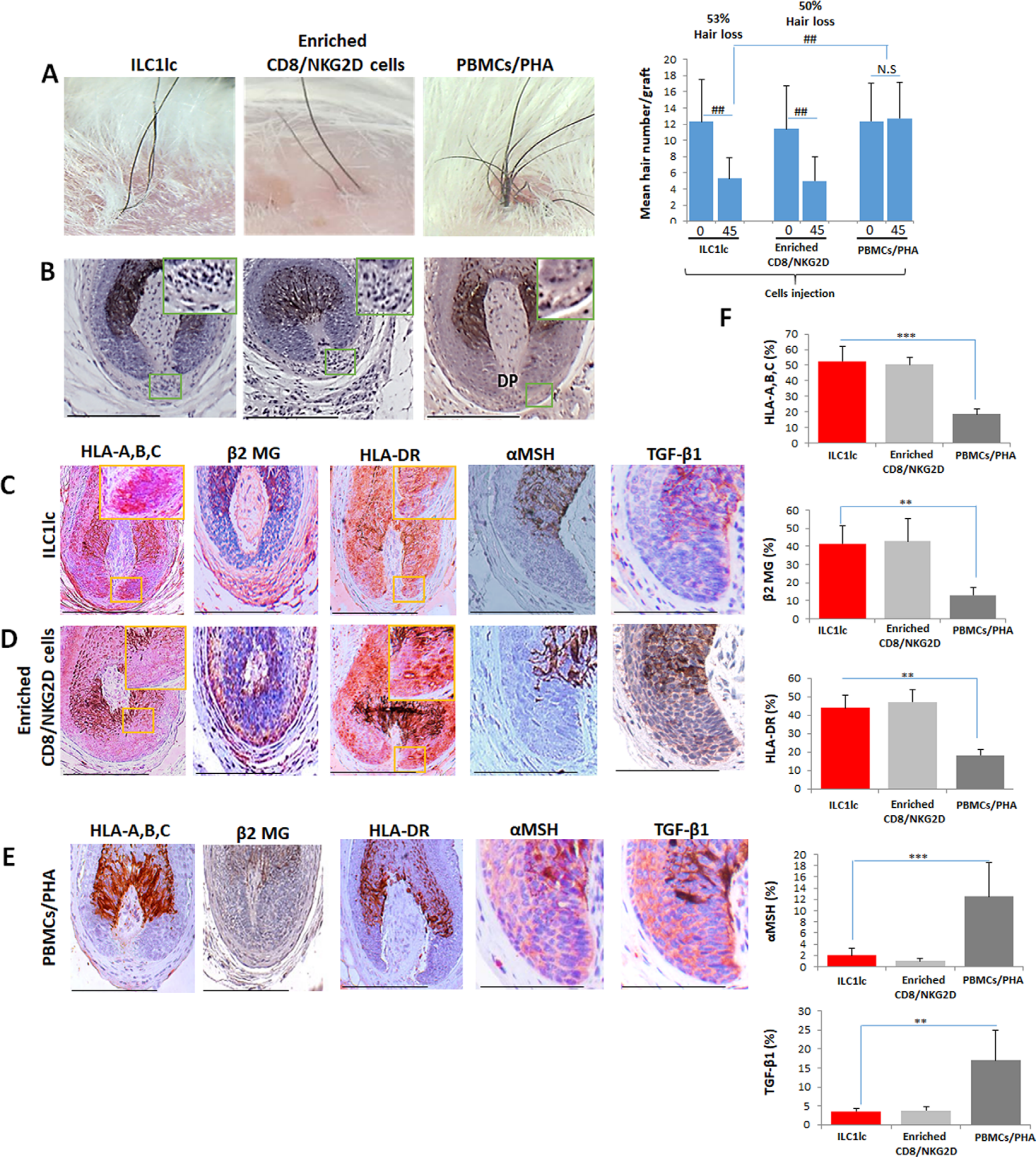
Development of alopecia areata (AA) in the humanized mouse model treated with ILC1lc. (**A**) Significant hair loss is observed following the injection of ILC1lc and enriched CD8/NKG2D cells, while in the PBMCs/PHA treated group, hair number remains almost constant. N=7–9 xenotransplants/group from three independent donors, Following Shapiro-Wilk test, Mann-Whitney *U* test: ^#^p<0.05, ^##^p<0.01. (**B**) HF dystrophy and perifollicular lymphocytic infiltrates around anagen hair follicles (HFs) (H&E staining) combined with strong expression of (**C**) HLA-A,B,C, β2 MG, HLA-DR, and downregulation of α-MSH and TGF-β1 in the ILC1lc and in (**D**) enriched CD8/NKG2D cells versus xenotransplants treated with (**E**) PBMCs/PHA (IHC staining) (**F**) quantitative data. N=5–9 xenotransplats/group from three independent donors. 4–5 defined reference areas were evaluated per section, and three sections per xenotransplants. Following Shapiro-Wilk test, Student’s *t*-test: *p<0.05, **p<0.01, ***p<0.001. Scale bar, 50 µm. DP - dermal papilla, HM - hair matrix. Figure 7—source data 1.Quantitative data for development of AA in the humanized mouse model treated with ILC1lc.

For this, 10 SCID/beige mice were each xenotransplanted with three full-thickness human scalp skin grafts (3 mm) obtained from parietal skin regions of four healthy donors without a prior history of AA (males aged 37±6). Eighty-nine days after transplantation, that is when hair regrowth had occurred in all xenotransplants, the mice were randomly divided into three groups, and each mouse from each group received one intradermal injection of either autologous IL-12/IL-18/IL-33-preactivated ILC1lc (test), PBMCs co-cultured with a nonspecific mitogen (PHA; negative control), or enriched CD8/NKG2D cells (positive control). When measured 45 days later, significant AA-like hair loss was observed macroscopically in the xenotransplants injected with ILC1lc compared to the negative control, at about the same level as positive control xenotransplants (Figure 7A).

**Figure 7—figure supplement 1.**
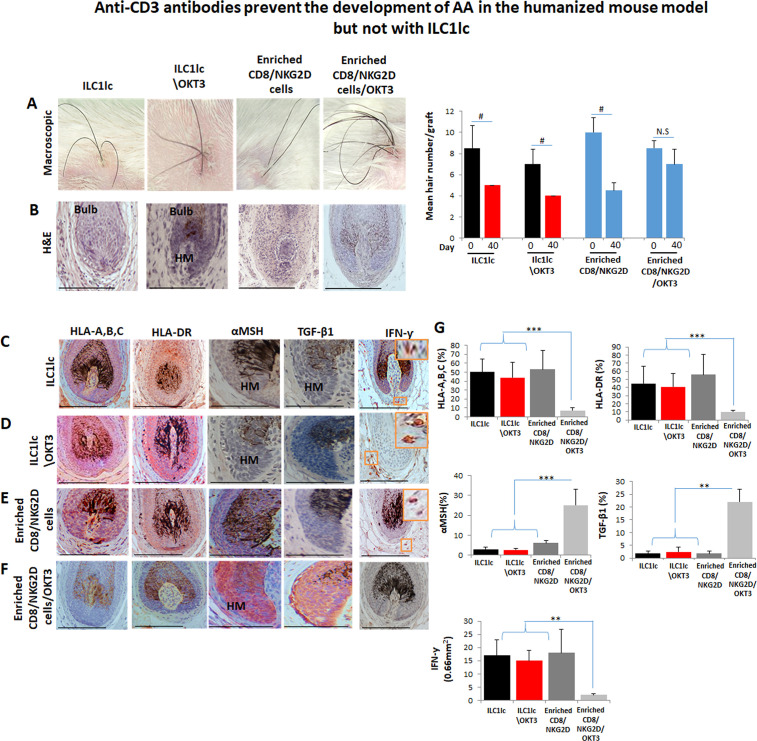
Anti-CD3 antibodies prevent the development of alopecia areata (AA) in scalp skin xenotransplants treated with enriched CD8/NKG2D but not with ILC1lc. (**A**) Significant hair loss was observed following the injection of ILC1lc, ILC1lc /OKT3, or enriched CD8/NKG2D cells into normal scalp skin on SCID/beige mice, but not in those treated with enriched CD8+/NKG2D+/anti-CD3 (OKT3), N=5 xenotransplants/group from two independent donors. Following Shapiro-Wilk test, Mann-Whitney *U* test: ^#^p<0.05, ^##^p<0.01. Hair follicle (HF) dystrophy combined with perifollicular lymphocytic infiltrate around anagen HFs (H&E staining) in xenotransplants treated with (**B**) ILC1lc, ILC1lc /OKT3, or enriched CD8/NKG2D. Normal HF in anagen in xenotransplant treated with enriched CD8/NKG2D/OKT3. Induction of HLA-A,B,C and of HLA-DR, downregulation of α-MSH and TGF-β1 by the follicular epithelium and increased dermal IFN-γ cells (IHC staining) in xenotransplants treated with (**C**) ILC1lc and similarly in (**D**) ILC1lc /OKT3 and (**E**) enriched CD8/NKG2D treated groups. Reduced expression of HLA-A,B,C, HLA-DR, and upregulation of α-MSH and TGF-β1 combined with decreased number of dermal IFN-γ cells were observed in xenotransplants treated with (**F**) enriched CD8/NKG2D/OKT3 cells. (**G**) Quantitative data, N=5–6 xenotransplants/group from two independent donors. 3–4 areas were evaluated per section, and three sections per xenotransplant. Following Shapiro-Wilk test, Student’s *t*-test: *p<0.05, **p<0.01, ***p<0.001. Scale bar, 50 µm. HM - hair matrix. Figure 7—figure supplement 1—source data 1.Quantitative data for anti-CD3 antibodies prevent the development of AA in scalp skin xenotransplants treated with enriched CD8/NKG2D but not with ILC1lc.

**Figure 7—figure supplement 2.**
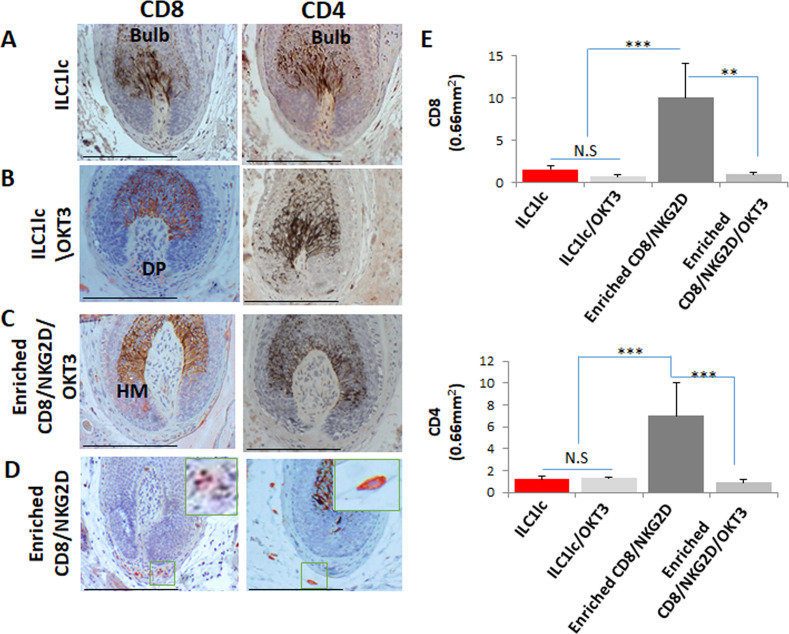
Dermal infiltrates of the various treated xenotransplants. Several CD8 and CD4 in xenotransplants treated with (**A**) ILC1lc and similarly in xenotransplants treated with (**B**) ILC1lc /OKT3, and (**C**) enriched CD8/NKG2D/OKT3 cells versus increased CD8 and CD4 in xenotransplants treated with (**D**) enriched CD8/NKG2D cells. (**E**) Quantitative data,N=5–6 xenotransplants/group from two independent donors, 3–four areas were evaluated per section, and three sections per xenotransplant. Following Shapiro-Wilk test, Student’s *t*-test: *p<0.05, **p<0.01, ***p<0.001. Scale bar, 50 µm. DP - dermal papilla,HM - hair matrix. Figure 7—figure supplement 2—source data 1.Quantitative data for dermal infiltrates of the various treated xenotransplants.

**Figure 7—figure supplement 3.**
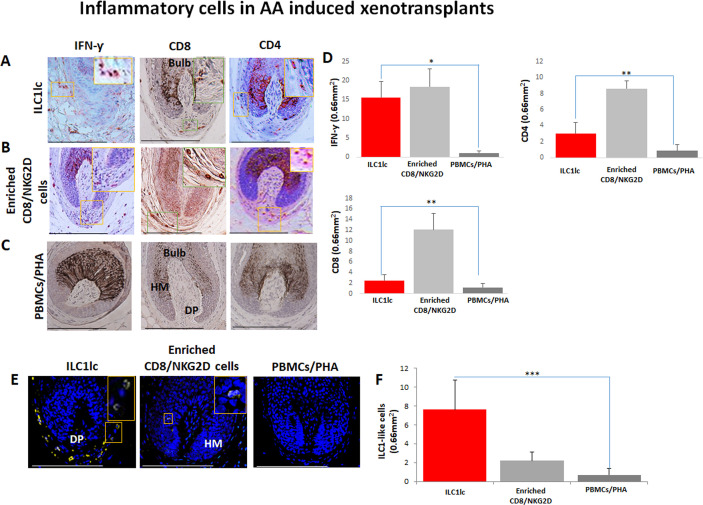
Development of alopecia areata (AA) in normal human scalp skin xenotransplants treated with (**A**) ILC1lc. Infiltrates of IFN-γ +, CD8, and CD4 cells in group treated with (**B**) enriched CD8/NKG2D cells. Decreased number of IFN-γ +, CD8, and CD4 cells in the (**C**) PBMCs/PHA treated one. (**D**) Quantitative data (**E**) presence of ILC1lc in transplants injected with ILC1lc compared to the virtual absence of these cells in enriched CD8/NKG2D and PBMCs/PHA treated mice.(**F**) Quantitative data N=5–9 xenotransplats/group from three donors. 4–5 areas were evaluated per section, and three sections per xenotransplant. Following Shapiro-Wilk test, Student’s *t*-test: *p<0.05, **p<0.01, ***p<0.001. Scale bar, 50 µm. DP - dermal papilla,HM - hair matrix. Figure 7—figure supplement 3—source data 1.Quantitative data for development of AA in normal human scalp skin xenotransplants treated with ILC1lc, enriched CD8/NKG2D and PBMCs/PHA.

To exclude that the above phenomena were not caused by residual human T-cells present in the transplants, an additional eight xenotransplanted mice were also injected intradermally once daily for 45 days with anti-CD3 antibodies (OKT3), in addition to injecting either ILC1lc or enriched CD8/NKG2D cells as described above (four mice each). This showed that anti-CD3 failed to abrogate hair loss induction in the mice treated with ILC1lc alone, but suppressed hair loss in the group treated with enriched CD8/NKG2D cells, as expected (Figure 7—figure supplement 1A). These findings invalidate the residual T-cell hypothesis.

### ILC1lc induce the characteristic immunopathology of human AA lesions in vivo

Immunohistology revealed that ILC1lc, just like autologous enriched CD8/NKG2D cells, induced a phenocopy of AA immunopathology in previously healthy human scalp skin in vivo, in sharp contrast to the negative control PBMCs/PHA group: HFs dystrophy, miniaturization and perifollicular lymphocytic infiltrate around anagen HFs (Figure 7B) as well as induction of HF-IP collapse (significantly increased expression of HLA-A,B,C, β2 MG, and HLA-DR of the HF epithelium, along with downregulation of the immune privilege guardians, α-MSH and TGF-β1) (Figure 7C and D). In contrast, negative control xenotransplants injected with PBMCs/PHA showed normal anagen HFs and a significantly lower expression of HLA-A,B,C, β2 MG, and HLA-DR, paired with the expected normal expression levels α-MSH and TGF-β1 protein (Figure 7E) as assessed by qIHM (Figure 7F).

In addition, histology and quantitative immunohistomorphometry confirmed the preventive effect of anti-CD3 antibodies in inducing an AA-like phenotype in xenotransplants treated with enriched CD8/NKG2D cells, but not in those treated with ILC1lc (Figure 7—figure supplement 1B,C,D,E,F and G and Figure 7—figure supplement 2).

In line with the key role of IFN-γ in the development of AA (Gilhar et al., 2012; Gilhar et al., 2019a), IFN-γ+ cells were found to be increased around the bulb of xenotransplants injected with ILC1lc, even in the presence of the anti-CD3 antibody (OKT3), or with enriched CD8/NKG2D cells, but not with PHA-treated PBMCs or enriched CD8/NKG2D cells in the presence of OKT3 (Figure 7—figure supplement 1A,B,C,D,E,F and G and Figure 7—figure supplement 2A,B,C and D).

### ILC1lc in the experimentally induced AA lesions

Given that both, enriched CD8/NKG2D cells, and ILC1lc produce high amounts of IFN-γ, we then investigated the subtype of these cells around the bulb of control and treated xenotransplants, along with the frequencies of CD4 +T cells. In enriched CD8/NKG2D cells injected xenotransplants, the number of CD8 + cells and CD4 +T cells was significantly increased as compared to ILC1lc (p<0.001, p<0.001) (Figure 7—figure supplement 3A,B,C and D) while dense infiltration of ILC1lc was found only in xenotransplants treated with the purified ILC1lc (Figure 7—figure supplement 3E and F).

**Figure 8.**
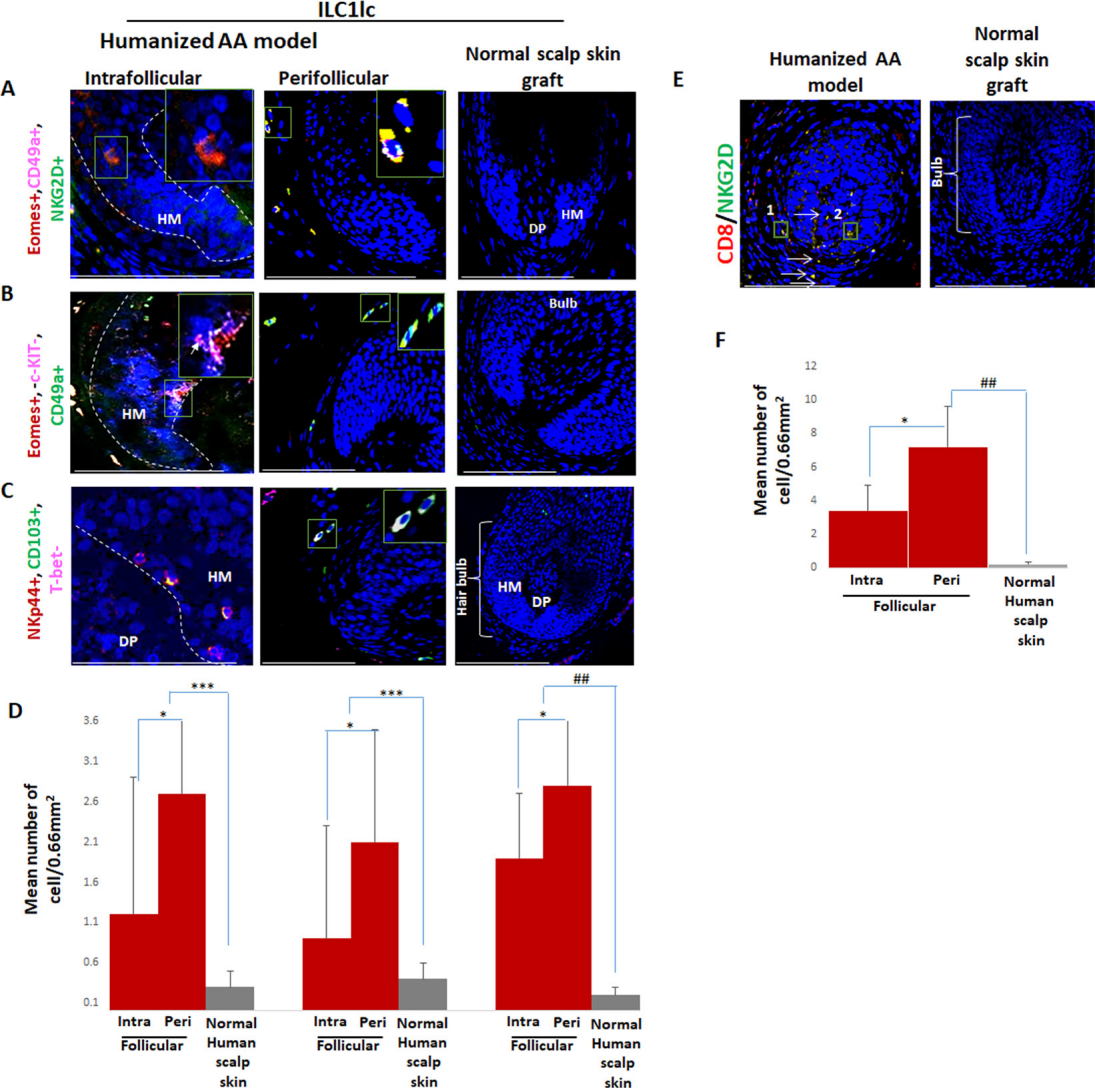
Immunofluorescence microscopy analysis of ILC1lc and enriched CD8/NKG2D cells in AA-induced xenotransplant. (**A**) EOMES+, CD49a+, and NKG2D+ around hair follicle (HF) in normal scalp skin, intrafollicular and perifollicular ILC1lc infiltrates in AA-induced xenotransplants (**B**) EOMES+,c-KIT-,CD49a+, and (**C**) NKp44+, CD103+, T-bet- ILC1lc. Absence of these cells in normal scalp xenotransplant. For each panel, yellow staining indicates double staining A-EOMES+, NKG2D+; B- EOMES+,CD49a+; C- NKp44+, CD103+. (**D**) Quantitation. (**E**) CD8+/NKG2D+ cells around HF in AA-induced xenotransplant versus absence of the cells in normal xenotransplant. (**F**) The quantitative data demonstrate the significant increased CD8+/NKG2D+ cells in HFs of alopecia areata (AA) humanized mice compared to normal scalp xenotransplants. N=6 xenotransplants/ group from three independent donors, three areas were evaluated per section. Following Shapiro-Wilk test, Student’s *t*-test: *p<0.05, **p<0.01, ***p<0.001. Mann Whitney *U* test: ^#^p<0.05, ^##^p<0.01.Scale bar, 50 µm. DP - dermal papilla, HM - hair matrix, White arrow- c-KIT stained melanocyte. Figure 8—source data 1.Quantitative data for immunofluorescence microscopy analysis of ILC1lc and enriched CD8/NKG2D cells in AA-induced xenotransplant.

Interestingly, qIHM also showed that the peri- and intrafollicular distribution and mean number of ILC1lc in human skin xenotransplants injected with enriched CD8/NKG2D cells imitated that of ILC1lc seen in spontaneously developed hair loss lesions of AA patients, further supporting the role of ILC1lc in human AA (Figure 1A, B, C and D, Figure 8A, B, C and D and Figure 1—figure supplement 1B). Yet, CD8+/NKG2D+lymphocytes significantly outnumbered ILC1lc in the experimentally induced AA lesions (p<0.01) (Figure 8E and F), just as they do in human AA patients (Figure 1E and F).

## Discussion

The current study is the first to phenotypically and functionally explore the role of Eomes +ILC1 lc in human AA in vivo and ex vivo. Eomes +ILCs may represent the NK cell lineage and therefore the ILC1 phenotype in our study, more closely resembles a tissue-resident or activated NK cell rather than an ILC1, based on recent single-cell RNAseq studies in mice and human tissues (Lopes et al., 2023). However, Eomes expression by ILCs was observed differently depending on the tissue localization (McFarland et al., 2021). For example, a recent single-cell RNA-sequencing study reveals that ILC1lc both in blood and tonsil are Eomes positive (Mazzurana et al., 2021). Other study demonstrated that intraepithelial ILC1s from human tonsils were found to be heterogeneous, encompassing Eomes− and Eomes +subsets (Cella et al., 2019). It is indeed very important to obtain greater clarity on how ILC1lc and NKs differ from each other, namely in the context of human AA. Our new data demonstrate that ILC1lc are positive for CD49a, CD200R, CD127, CXCR6 (Lopes et al., 2023) but negative for IRF8, Perforin, NKp80, CD16 (Sagebiel et al., 2019; Figure 2—figure supplement 1A,B and C), strongly support that ILC1lc and NK cells have distinct lineages. This claim remains limited by the set of biomarkers that is currently available (and can reasonably be expected to be applied) to ‘definitively’ distinguish between ILC1 and NK lineages, namely in human skin, and that our data may eventually have to be re-evaluated in the context of research progress in this fast-moving field. It would be desirable to further complement these results in future studies with unbiased scRNAseq data, which can then be compared with published human ILC1 and NK cell gene signatures so as to gain deeper insights into the – still controversial and unclear - transcriptional similarities and differences between ILC1lc and NK cells and their lineage relationship to each other.

Here, we show that ILC1lc are increased in AA lesions provide the first functional evidence that expanded circulating autologous human ILC1lc suffice to induce all hallmarks of the AA hair loss phenotype (premature catagen, HF dystrophy, and HF-IP collapse) in previously healthy, organ-cultured human scalp HFs ex vivo and in human scalp skin xenotransplants in vivo, where they also cause the characteristic clinical hair loss phenomenon. This also provides the first unequivocal functional evidence of a key role of ILC1lc innate lymphocytes in a model human autoimmune disease, and thus identifies these lymphocytes as important novel targets in future AA therapy.

**Figure 9.**
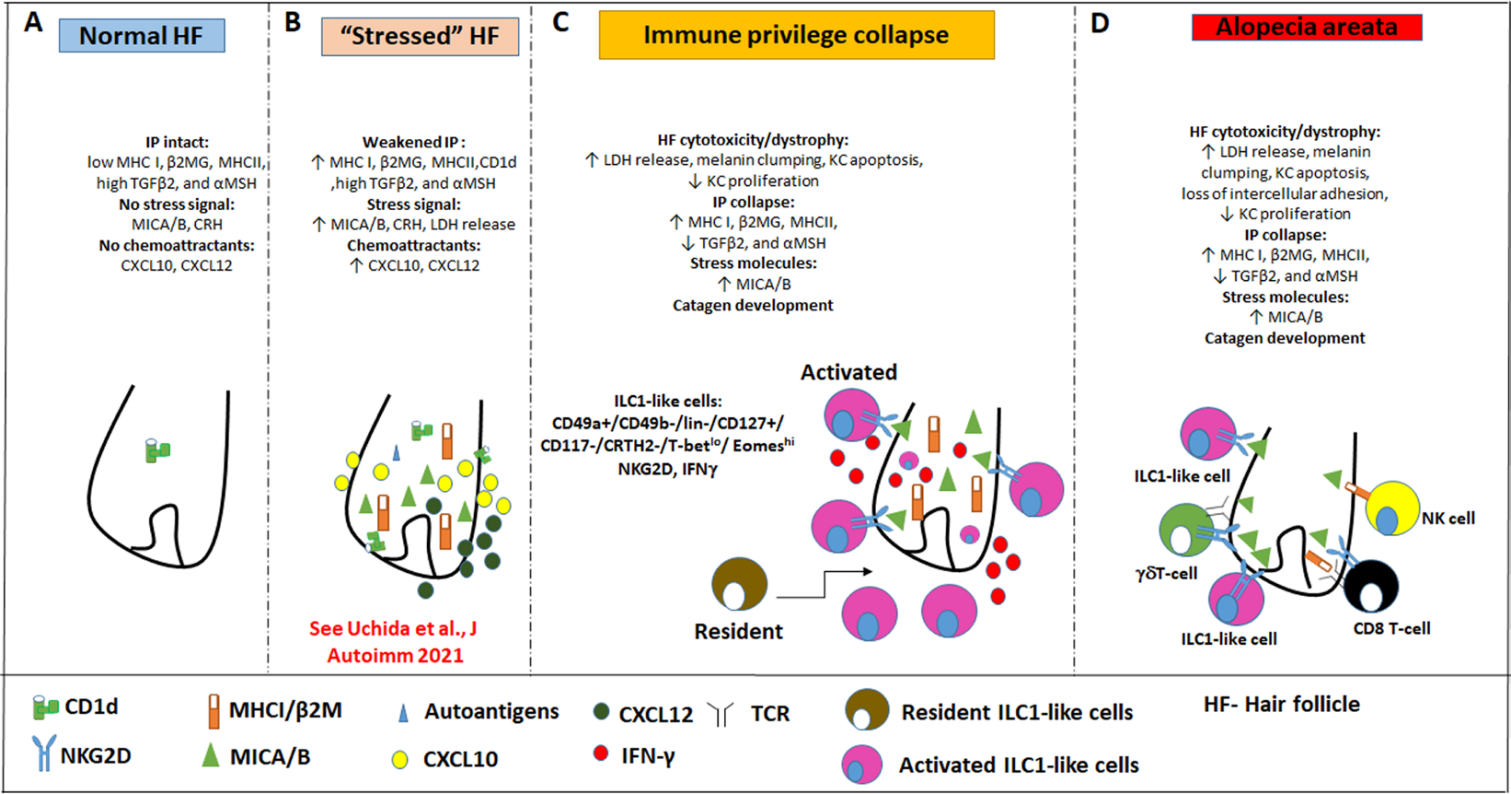
Pathobiology scenario: How ILC1lc can induce alopecia areata (AA). (**A**) ILC1lc are rarely detected around the bulb of healthy human scalp hair follicles (HFs), which exhibit relative immune privilege and low or absent expression of MICA and MHC class I, and CD1d. (**B**) Various tissue stressors (in the current study: hair follicle microdissection and organ culture), can transiently weaken the hair follicle’s physiological immune privilege by upregulating the expression of MHC class I, MICA (a key activating NKG2D ligand), and of CD1d, along with the secretion of chemoattractants such as CXCL12. (**C**) This recruits and activates ILC1lc, which migrates towards the ‘stressed’ hair follicle and secretes IFN-γ, thus ultimately inducing HF-IP collapse. (**D**) Either alone or in conjunction with other recognized AA-inducing immune cells (i.e. CD8 +T cells which recognize hair follicle autoantigens now exposed by ectopically expressed MHC class I; NK cells, and *γδ*TCs), ILC1lc can then induce the full AA phenotype, characterized by HF-IP collapse, premature hair follicle regression (catagen), and hair follicle dystrophy.

Mechanistically, we demonstrate that IFN-γsecretion and NKG2D signaling are both required for this AA-pattern HF damage to occur (Figure 9). That ILC1lc alone can induce all hallmarks of AA in a healthy human (mini-) organ ex vivo and in vivo, presumably in an autoantigen-independent manner, also demonstrates that these innate/transitional lymphocytes interact directly with human HFs, rather than affecting them only indirectly. We also show that resident T-cells in human scalp skin transplants are not responsible for the AA-inducing effects of ILC1lc in vivo (Figure 7—figure supplement 1 and Figure 7—figure supplement 2).

Our study demonstrates that CD8^+^ T cells, which have long been thought to represent the central players in AA pathobiology (Pratt et al., 2017; Xing et al., 2014; de Jong et al., 2018; Gilhar et al., 1998; Wang et al., 2016; Paus et al., 1993), are not the only drivers of disease (Gilhar et al., 2019a; Pratt et al., 2017; de Jong et al., 2018; Paus et al., 1993), and are no joined not only by NK cells (Ito et al., 2008; Gilhar et al., 2013a) and γδT cells (Uchida et al., 2020; Uchida et al., 2021), but also by ILC1lc lymphocytes. The study further supports the concept that the characteristic hair loss pattern we diagnose as AA phenotype, does not always represent a classical, autoantigen- and autoreactive CD8+/NKGD2 +T-cell-dependent autoimmune disease (‘autoimmune AA’ [AAA]), but can also reflect non-autoimmune pathomechanisms that may perhaps best be defined as non-autoimmune AA subtype (NAIAA) (Bertolini et al., 2020; Paus et al., 2018; Figure 9). In a state of prolonged HF-IP collapse and thus chronic exposure of HF-associated autoantigens to (pre-existent?) autoreactive CD8 +T cells, it is well conceivable that an AA-subtype that began as NAIAA can over time transform into the AAA-variant, thus explaining the chronic-intermittent course that is seen in so many autoimmune diseases.

The novel concept of ILC1-induced NAIAA mandates a differential, personalized management approach to future AA therapy, which tailors treatment to the specific pathobiology at hand in any given AA patient. This must now include the targeting of pathogenic, potently IFN-γ -secreting ILC1lc, at least when these are seen to be increased in lesional AA biopsies.

Although the number of ILC1lc in lesional AA skin was significantly lower than that of CD8 + cells (Figure 1) this does not rule out a crucial role of the cells in spontaneous AA development in patients. In fact, we demonstrate here that ILC1lc are even more potent IFN-γproducers than CD8 +T cells. Also, despite the relatively low numbers of ILC1lc, their selective tissue distribution makes them ideally localized to provide an early source of cytokines to initiate/trigger pro-inflammatory immune responses directed against distressed tissues (Vivier, 2021), as documented here in our co-culture assay with ‘stressed’ human scalp HFs ex vivo.

That IL-12 and IL-18 were among the cytokines used here to facilitate ILC1lc isolation from human PBMCs is also interesting in the context of our most recent observation that local IL-12 signaling, supported by IL-18, may be involved in the early stages of AA development by stimulating IFN-γ production from resident IL-12Rß2+immune cells, eventually leading to HF-IP collapse (Edelkamp, 2021). In fact, treatment with IL-12 +IL-18 of healthy HFs selectively enriches IFN-γ -inducible genes and promotes the release of IFN-γ into the medium and thus HF-IP collapse. These responses were abrogated by the co-administration of a selective TYK2 inhibitor (Edelkamp, 2021), which confirms a key role of IL-12, whose receptor utilizes a TYK2 and Janus kinase 2 pair for downstream signal transduction (Ullrich et al., 2020; Krueger et al., 2022), These preliminary findings suggest that IL-12 is a key effector cytokine in promoting pathogenic IFN-γ secretion and HF-IP collapse. Given that ILC1 are IFN-γ producing cells (Nabekura and Shibuya, 2021b; Resende et al., 2017; Quintino-de-Carvalho et al., 2022), our data, therefore, encourage one to dissect, next, the exact role of IL-12/IL-12R-mediated signaling in activating, attracting and/or expanding ILC1lc and in stimulating their IFN-γ secretion in the early stages of human AA pathogenesis.

An immunopathology-initiating role of ILC1lc is not unique to AA and has also been proposed in other inflammatory conditions (Kim et al., 2021), including vitiligo (Tulic et al., 2019), which shares some pathogenesis features with AA (Harris, 2013; Tomaszewska et al., 2020), inflammatory bowel disease (McDonald et al., 2018; Bernink et al., 2013; Tang et al., 2019), lupus erythematosus (Guo et al., 2019), and the aggravation of atherosclerosis (Wu et al., 2018). Yet, to the best of our knowledge, the current study is the first to demonstrate that these innate immunocytes can indeed induce the full disease-mimicking immunopathology phenotype in a previously healthy human (mini-) organ. Also, abnormalities in the crosstalk between ILC1lc and gut microbiota have been observed in various diseases (Jiao et al., 2020). Therefore, it is interesting to ask whether the microbial dysbiosis that has been reported in the scalp skin of AA patients (Pinto et al., 2020) may activate the very few, strategically positioned perifollicular ILC1lc present in healthy human scalp skin through abnormal crosstalk with HF microbiota (Lousada et al., 2021). This hypothesis can now be explored using our ex vivo and humanized AA mouse model.

Collectively, our study introduces IFN-γ -secreting ILC1lc lymphocytes as important novel players in human AA pathobiology and identifies them as new therapeutic intervention targets. Their strategic location, their capability to recognize and respond to HF distress signals such as MICA and selected chemokines, the excessive production of IFN-γ by ILC1lc, and their direct, pathogenic effects on human HFs ex vivo documented here, all support that these cells play a hitherto unappreciated role in early AA pathogenesis. Moreover, our study demonstrates that autoreactive CD8 +T cells are not indispensable for AA induction and further supports that non-autoimmune AA variants (NAIAA) (Bertolini et al., 2020) exist and that innate/transitional immune cells play an important role in AA pathobiology.

## Materials and methods

### Patients, tissue, and blood samples

For the in situ analyses we used archival paraffin-embedded biopsy specimens of human AA scalp skin lesions from the Department of Pathology, Rambam Medical Center ( fourfemales, 12–35 years, mean age 20.5±9.5; sixmales, 6–38 years, mean age 18±12). Three of these AA patients showed active hair loss of the AA universalis phenotype while the other patients showed stable hair loss patches of the multifocal AA phenotype (Gilhar et al., 2012). One ten-year-old male patient had a positive family history of allergic rhinitis. AA was diagnosed both clinically and by histopathology, and none of the enrolled patients showed clinical evidence or had a personal history of other AA-associated autoimmune diseases (Gilhar et al., 2012; Meah et al., 2021).

Clinically healthy human skin scalp specimens were obtained from healthy volunteers undergoing cosmetic facelift surgery ( three females, 43–72 years, mean age 58±15; 5 males, 31–44 years, mean age 36±8).

The ex vivo experiments utilized scalp HFs from 20 healthy donors (13 males and seven females) without a history of AA (31–63 years, mean age 49±12).

The in vivo experiments in the humanized AA mouse model Ghraieb et al., 2018 used scalp skin pieces obtained from five healthy donors ( four males and one female, 34–45 years, mean age 38±5). For the ex vivo and in vivo experiments, frontotemporal human scalp skin specimens were obtained during elective cosmetic facelift procedures performed under general anesthesia, and 20 ml of autologous venous blood was drawn, both with informed written patient consent.

The study for both ex vivo and in vivo experiments was approved by the Institutional Ethics Committee of the Rambam Health Care Campus, Haifa, Israel (RMB-0182–14).

For the ex vivo experiments, frozen HFs sections were dehydrated for 40 min and incubated with acetone for 10 min at –20 °C for fixation. Slides were dehydrated for 20 min and transferred to double-distilled water following three times wash with 1xphosphate buffered saline (PBS) (pH = 7.4). Sections were blocked with suitable serum (horse/goat) for 30 min to prevent nonspecific binding and incubated at 4 °C with primary Ab overnight. Slides were incubated with an appropriate biotinylated secondary Ab (FITC-conjugated goat anti-mouse Ab, Rhodamine-conjugated goat anti-mouse IgG Ab or Alexa Fluor 488-conjugated goat anti-rabbit IgG Ab) for 30 min, following three times wash with PBS.

For the in vivo experiment, five-micrometer paraffin sections were used. Antigen retrieval was for 20  min at 90 °C in a microwave. Specimens were blocked for 30  min to prevent nonspecific binding and incubated with the first antibody (Ab) overnight, followed by a wash and incubation with biotinylated 2^nd^ Ab (Jackson ImmunoResearch, West Grove, PA), and subsequent binding with horseradish peroxidase-conjugated streptavidin. Markers were revealed with AEC (red) (Aminoethyl Carbazole Substrate kit). Sections were then mounted and analyzed under a light microscope.

### Histochemistry, immunohistology, and quantitative immunohistomorphometry (qIHM)

Five-micrometer paraffin sections of lesional AA biopsies and human scalp skin xenotransplants were processed for histochemistry or immunohistology. The following primary antibodies were used: anti-CD8 (Cell Marque-108M-95), anti-CD4 (DAKO-M7310), anti-HLA-A,B,C (Abcam-70328), anti-HLA-DR (Abcam-20281), anti- IFN-γ (Abcam-25101), anti- α-MSH (LSBio-C25584), anti-beta2 microglobulin (Abcam-218230), and anti-TGF-β1 (Santa Cruz-52893) (Ghraieb et al., 2018; Laufer Britva et al., 2020; Keren et al., 2018).

Since there is no single, highly specific surface marker for ILC1 cells, triple immunostaining was performed with three sets of antibodies that are routinely used for the identification of human ILC1lc (Talayero et al., 2016; Hawke et al., 2020a; Hawke et al., 2020b; Cruz-Zárate et al., 2018): (a) NKp44+ (Bioss-YEYS3W) (Talayero et al., 2016), CD103+ (eBioscience 1401038–82) (Hawke et al., 2020b) and T-bet- (Santa Cruz-H3112) Cruz-Zárate et al., 2018; (b) c-KIT- (DAKO-MA512944) (Nagasawa et al., 2019), CD49a+ (R&D systems-AF5676) (Colonna, 2018) and EOMES+ (ThermoFisher-14-4877-82); and (c) CD49a+ (R&D systems), EOMES+ (ThermoFisher-14-4877-82) and NKG2D+ (Novus-5c6) (de Jong et al., 2018). Skin-infiltrating CD8 +NKG2D+T cells were double-immunostained by NKG2D (Novus-5c6)/CD8 (Cell Marque-108M-95) (de Jong et al., 2018). For negative control, the primary antibody was replaced with non-specific IgG1 and IgG2 isotype control.

Hematoxylin and eosin (H&E) staining was performed on cryo- or paraffin sections as previously described (Keren et al., 2018). For the ex vivo experiments, HFs cryosections were dehydrated for 40 min and fixed with acetone for 10 min at –20 °C (Ghraieb et al., 2018; Laufer Britva et al., 2020; Keren et al., 2015).

The following primary antibodies were used for immunohistochemistry (IHC) or immunofluorescence microscopy (IF) of key HF immune privilege markers (Bertolini et al., 2020; Paus et al., 2005) anti-HLA-A,B,C (Abcam-70328)/ anti-HLA-DR (Abcam-20281)/anti-MICA(Santa Cruz- 20931)/anti-CD1d (Abcam-11076)/anti- α-MSH (LSBio-C25584)/anti-beta2 microglobulin (Abcam-218230) and anti-TGF-β1(Santa Cruz-52893).

The immunoreactivity patterns were assessed in standardized, well-defined reference areas by quantitative immunohistomorphoemtry (qIHM) by experienced, blinded observers, following our standard protocols for evaluating human HF immunology read-outs (Bertolini et al., 2014; Bertolini et al., 2016; Christoph et al., 2000; Harries et al., 2013; Hardman-Smart et al., 2020), counting at least three reference areas each on three non-consecutive sections, presented randomly to the blinded observer(s). Specifically, immunoreactive cells around and within the HFs were counted in an area of 0.66  mm^2^.

For HLA-A,B,C, HLA-DR, MICA, CD1d, α-MSH, beta2 microglobulin, and TGF-β1 image analysis was performed using Image J software. Protein expression was measured by calculating the percentage of staining coverage within the analyzed area.

Masson-Fontana staining (Abcam) was performed as described by us (Laufer Britva et al., 2020; Purba et al., 2016). Briefly, five-micrometer paraffin sections were deparaffinized and hydrated in distilled water. Slides were placed in mixed ammoniacal silver solution in a 58–60°C water bath and allowed adequate time for the temperature to equilibrate. Slides were then placed in the warmed ammoniacal silver solution for 30–60 min or until the tissue section became yellowish/brown in color. Counterstaining was performed with Nuclear Fast Red Solution for 5 min.

### TUNEL analysis

Apoptotic cells were evaluated using a commercial TUNEL kit (Roche) with anti-digoxigenin fluorescein labeling and according to the manufacturer’s protocol. Ki-67 (Invitrogen) was visualized using Alexa Flour 594-conjugated goat anti-mouse (Jackson, 115-585-062). Sections were counterstained by DAPI (Thermo Fisher Scientific). Staining was visualized using a confocal Microscope - Zeiss LSM 700. Quantification was performed as previously described (Peters et al., 2006).

### Immunohistology

Slides were photographed using immunofluorescence confocal microscopy and compared systematically by qIHM in standardized, defined tissue compartments. Mouse skin served as a negative control. Three non-consecutive sections were analyzed per patient.

### Isolation, characterization, and culture of circulating ILC1lc, ILC2, ILC3, and CD8+/NKG2D+ cells

ILC2 and ILC3 cells were used as negative controls, while CD8+/NKG2D+ cells were used as a positive control to evaluate the ILC1lc cytotoxic effects on HFs.

The cells were cultured and induced to expand, as we have previously described (Keren et al., 2018). The isolation and characterization of ILC1lc, ILC2, and ILC3 cells by FACS cell sorting or MACS were performed as we previously described (Keren et al., 2018; Mjösberg et al., 2011; Mora-Velandia et al., 2017; Creyns et al., 2020).

Autologous human PBMCs were isolated from heparinized whole blood from healthy donors by Lymphoprep density gradient centrifugation (Alere Technologies, Norway). Cells were frozen for further assays (70% FBS, 20% RPMI1640, and 10% DMSO) or cultured at a seeding density of 3 × 10^6^ cells/ml in 24 wells plate with medium (RPMI 1640, 10% human AB serum, 1% penicillin-streptomycin antibiotics, 2 mML glutamine) in the presence of different cytokines for cells expansion.

The following components are required for the expansion of various immune cell populations:

ILC1lc (Silver et al., 2016): IL-18(1 µg/1 ml) (CYT-269(A), IL-33) (1.5 µg/5 ml) (BLG-581802), IL-12 (1.5 µg/5 ml) (BLG-573002).
ILC2 (Creyns et al., 2020): IL-7 (10 ng/ ml) (BLG-581904), IL-25 (100 ng/ml) (C792-50), IL-2 (50 ng/m) (Prospec-Cyt-209-b).
ILC3 (Keren et al., 2018): AHR (200 nM) (BML-GR2060100), IL-2 (100 U/mL).
CD8 +NKG2D+ (Gilhar et al., 2013a): IL-2 (100 U/ml).
PHA (Gilhar et al., 2013a): PHA (10 µg/ml) (Sigma-C1668).

On days three and five, half of the medium was either frozen for further analysis or discarded and replaced with fresh medium containing cytokines. After seven days, cells were sorted by FACS Aria (FACSAria III Cell Sorter, BD Biosciences, USA) and in the case of ILC3 further enriched by MACS for negative selection of CD3 + cells (see Figure 2—figure supplement 2).

### Flow cytometry sorting for ILC1lc, ILC2, and CD8+/NKG2D+ cells

Cells were cultured for one week, collected, and washed with PBS containing 1% BSA and 2% PSN. Surface cells were stained with antibodies to the PE-conjugated lineage cocktail that includes antibodies against CD1a (BLG-300105), CD3 (BLG-300–307), CD14 (BLG-367103), CD19 (BLG-302207), CD34 (BLG-343605), CD123 (BLG-306005), CD11c (BLG-301605), BDCA2 (BLG-354203), FcεR1α (BLG-334609), TCRαβ (BLG-306707), TCRγδ (BLG-331209), CD56 (BLG- 362565) (Hawke et al., 2020b).

Following gating on lineage, cell population cells were sorted as follows:

ILC1lc – APC-conjugated-CD127+ (BLG-351315), PE/CY7-conjugated-CD161+ (BLG-339917), and FITC-conjugated-NKp44 + (SC-53597), Brilliant Violet 421TM anti-human CD117- (c-KIT) (BLG-313215) and APC/CY7-conjugated-CRTH2- (BLG-350113). (Santa Cruz H3112) (Talayero et al., 2016; Hawke et al., 2020a).

ILC2 cells – APC-conjugated-CD127+ (BLG-351315), PE/CY7-conjugated-CD161+ (BLG-339917), Brilliant Violet 421TM anti-human CD117+ (c-KIT) (BLG-313215) and APC/CY7-conjugated-CRTH2+ (BLG-350113) (Cruz-Zárate et al., 2018; Nagasawa et al., 2019).

CD8+/NKG2D+ cells – CD8 (Cell Marque-108M-95)/NKG2D(Novus-5c6) (Ito et al., 2008). The cells were sorted using a FACS Aria instrument with software (BD Biosciences). The sorted cells were collected in a tube with a medium enriched with 20% human serum. Afterward, the cells were centrifuged, suspended, counted, and co-cultured with HFs or used for ELISA assay.

Compensation was done using Comp-Beads (BDTM Biosciences) and data were analyzed using FlowJo software.

### Magnetic isolation of ILC3 subsets

Separation was performed using anti-CD3 antibodies conjugated to ferromagnetic microbeads (Miltenyi Biotec, Bergisch Gladbach, Germany) and directed through a cell separation column containing a magnetic field (Miltenyi Biotec). For the purification of ILC3s, CD3−sorted cells were collected and stained with anti-NKp44-PE conjugated to ferromagnetic microbeads (Miltenyi Biotec) and directed through a cell separation column containing a magnetic field (Keren et al., 2018).

Finally, cells were co-cultured with autologous HFs ex vivo (see below) or used for different assays (Keren et al., 2018).

### Co-culture of autologous ILC1lc with ‘stressed’ human scalp hair follicles ex vivo

Experimental induction of HF-IP collapse by IFN-γ is the standard ex vivo-assay system for interrogating key elements of AA-related human HF immunopathology (Ito et al., 2004; Bertolini et al., 2016; Kinori et al., 2012). We have recently complemented this assay by co-culturing key immunocytes in AA pathogenesis (CD8 +T cells, γδTCs) directly with organ-cultured human scalp HFs ex vivo (Uchida et al., 2021).

For this, healthy human anagen scalp HFs were collected and microdissected as described (Ito et al., 2004), and HFs were placed individually into a 96-well plate slot with 100 µl supplemented medium (William’s E plus 1% penicillin-streptomycin antibiotics, 1% L-glutamine (Invitrogen-Gibco)), 0.01% hydrocortisone (Sigma-Aldrich) and 0.01% insulin (Sigma-Aldrich) (Langan et al., 2015).

As we have documented in detail elsewhere (Uchida et al., 2021), on day 1 after initiation of organ culture, the HFs are markedly, but transiently stressed by the trauma of microdissection and the transfer to a harsh, hyperoxygenated ex vivo culture environment. This results in significantly increased LDH activity release and up-regulation of CXCL12 and CXCL10 expression as well as in a transient, partial weakening of the HFs physiological immune privilege. The latter was evidenced by increased protein expression of MHC class Ia, β2-microglobulin, and MICA/B but no change in the expression of IP guardians such as αMSH and TGFβ2. As reported before, all these ‘HF stress’ indicators normalize on day 3 of organ culture (Supplementary file 1) (Taken from: Uchida et al., 2021). Thus, due to their expression of NKG2D ligands (MICA/B) CXCL12 and CXCL10 secretion, and transiently weakened HF immune privilege, the stressed (day 1) HFs can attract and interact with immune cells expressing NKG2D receptors and are primed to elicit anti-HF immune responses ex vivo (Uchida et al., 2021).

Therefore, organ culture-stressed day 1 HFs (1HF/well) were co-cultured in supplemented William’s E medium from day 1 until day 6 with one of five different immune cell populations: (1) ILC1lc (100 µl/600 cells per well), either alone or in combination with anti- IFN-γ antibody (10 µg/ml, R&D Systems, MAB285) or NKG2D neutralizing antibody (5 µg/ml, R&D Systems, MAB139-100); ILC1lc demonstrated cytotoxic effect on HFs with 600 cells per well, while (2) CD8/NKG2D cells demonstrated similar effect only with 100 µl/3500 cells per well. Therefore, 100 µl/3500 per well was used for the following control groups: (3) ILC2s cells; (4) ILC3s, or (5) PHA cultured PBMCs.

The medium was not replaced in order to avoid losing any immunocytes. Basic HF biology read-out parameters were assessed by evaluating the Ki-67/TUNEL ratio, values of LDH release, HF pigmentation, and hair shaft production in situ, all of which indicated that the HFs did not suffer major damage after 6 days of organ culture. At the end of the experimentation, the HFs were photo-documented and cryopreserved in optimal cutting temperature (OCT) blocks. Cytokine release into the culture medium by ELISA was analyzed as previously described (Zook and Kee, 2016).

### Flow cytometry analysis for characterization of ILC1lc

PBMCs were isolated from healthy blood via centrifugation on ficol/Hypaque and cultured for seven days in a medium composed of RPMI 1640, 10% human AB serum, 1% L-glutamine, and 1% PSN. The medium was changed as needed.

Seven days later, 6 hr prior to FACS staining, cells were then collected (1–1.5 × 10^6^ cells/tube), centrifuged at 1200 RPM for 5 min, and washed twice in staining buffer (1 ml of 1% Bovine Serum Albumin [BSA] in 1 x sterile PBS). First antibodies (as described above, flow cytometry sorting) were used at a concentration of 2.5 μl per 1 × 10^6^ cells.

Cells were incubated for 25 min at room temperature in the dark. All tubes were washed once with 1 ml staining buffer, then Fixation/Permeabilization solution (250 μl) was added and cells were incubated for 20 min at 4 °C. Cell permeability was performed using 1 × BD Perm/Wash buffer, intra-cellular antibody mixtures (50 μl/Brilliant Violet 605TM anti-T-bet BLG 644817), Eomes-conjugated-PerCP-eFluor 710 (Dan11mag), IRF8 (sc-365042), Perforin (BLG-308119), and INF-γ-conjugated- PE/VIO-770 (Miltenyi Biotec 130-109-313), CD49a-APC-Vio770 (Miltenyi Biotec 130-101-324),FITC anti-human CD49b (BLG 359305), APC anti-human NKp80 (BLG 346707), BV421 anti-human CXCR6 (BLG 356013) and APC/Fire 810 anti-human CD16 (BLG 302073) and BV421 anti-human RORγt were added and incubated for 30 min at room temperature in the dark, cells were then washed twice with 1xBD Perm/Wash buffer (BD Cytofix/CytopermTM Fixation/Permeabilization Kit).

All cell samples were detected by FACS Calibur Flow Cytometer (Benton Dickinson) using Cell Quest software, and the acquired data were further analyzed using FlowJo 5.7.2 (Tree Star).

### Cytokine analyses in culture medium by ELISA

Production of IFN-γ by ILC1lc from healthy volunteers was analyzed using ELISA. ILC2, ILC3, and PBMCs/PHA were analyzed as negative controls. CD8+/NKG2D+ cells were analyzed as a positive control.

The concentration of IFN-γ was determined in the supernatant of 6 × 10^6^ cells from each donor (six healthy donors) using the Human IFN-γ ELISA deluxe set (BioLegend) according to the manufacturer’s protocol.

### Analysis of HF cytotoxicity, catagen induction, and immune privilege collapse

As an indication of HF cytotoxicity, LDH release into the supernatants was quantified by colorimetric assay using the Cytotoxicity Detection kit Plus (Roche), which measures the conversion of tetrazolium salt in formazan, a water-soluble dye with a broad absorption maximum at approximately 500 nm (Uchida et al., 2021; Lu et al., 2007; Poeggeler et al., 2010). Medium with/without HFs was cultured with PBMCs/PHA, CD8+/NKG2D+, ILC1lc, ILC2, or ILC3 cells for three days. Formazan absorbance was measured for each condition that correlates with cell cytotoxicity. Anagen and catagen HFs were visualized and differentiated under Nikon Diaphot inverted binocular and thereafter qualitative morphological and quantitative morphometric assessments were analyzed as previously described (Kloepper et al., 2010). IHC staining was performed to test all hallmarks of AA in order to probe whether co-culture with ILC1lc induced abnormal HLA-DR, HLA-ABC, CD1d, ß2-microglobulin, and MICA protein expression in the proximal HF epithelium and/or downregulated the key guardians of HF immune privilege, TGF-β1, and α-MSH (Bertolini et al., 2020; Ito et al., 2004), using the qIHM method described above.

In order to check whether ILC1lc affects HFs via IFN-γoverproduction or via activation of the NKG2D-NKG2DL axis following excessive MICA expression by stressed HFs, neutralizing anti- IFN-γ (10 µg/ml, R&D Systems, MAB285) or function-blocking NKG2D (5 µg/ml, R&D Systems, MAB139-100) antibodies, were added to the HFs co-cultured with ILC1lc (defined as CD49a+CD49b- Verma et al., 2020), lin-/CD127+/CD117-/CRTH2-, and T-bet^lo^/ Eomes^hi^ (Bennstein et al., 2020; Krabbendam et al., 2021).

### Humanized AA mouse model

For the humanized AA mouse model (Gilhar et al., 2013a; Ghraieb et al., 2018; Gilhar et al., 2016), full-thickness biopsies were taken from healthy donors undergoing plastic surgery on the scalp. Biopsies from each donor were dissected horizontally to generate pieces with a diameter of 3  mm. Three 3  mm pieces were grafted orthotopically into the subcutaneous layer of each SCID/beige mice as previously described (Gilhar et al., 2013a; Ghraieb et al., 2018; Gilhar et al., 2013b). Seven days after surgery, mice were treated with Minoxi-5 (hair regrowth treatment for men containing 5% Minoxidil active ingredient) by spreading it on the grafts twice a day until we received optimal expedited hair growth (period of two months). The topical minoxidil application is not required for hair regrowth induction after the initial post-transplantion hair shaft shedding, but only accelerates it. This application is discontinued before the ILC1lc injection (Gilhar et al., 2013a). Since the immune cell infiltrate in AA attacks only hair follicles in anagen (Gilhar et al., 2012), it is critical that the majority of xenotransplant HFs are in anagen at the time the immune cells are injected. Topical minoxidil pretreatment increases the likelihood that this is the case (Price et al., 1999; Suchonwanit et al., 2019). Moreover, we have recently demonstrated that 5% minoxidil does indeed significantly stimulate hair regrowth in human androgenetic alopecia scalp skin transplanted onto SCID/beige mice (Gilhar et al., 2022). It also deserves mentioning that topical minoxidil reduces the degranulation of – hair growth-modulatory! (Paus et al., 1994) - perifollicular mast cells in the skin of mice, namely under conditions of perceived stress (Arck et al., 2003), while excessive degranulation of perifollicular mast cells is an important feature of lesional human AA skin (Bertolini et al., 2014). Therefore, this pretreatment likely also helps to reestablish perifollicular mast cell homeostasis after the stress of xenotransplantation. In the current study, 18 female SCID/beige mice (C.B-17/IcrHsd-scid-bg) (Harlan Laboratories Ltd., Jerusalem, Israel) were used at 2–3 months of age and were housed in the pathogen-free animal facility of the Rappaport Faculty of Medicine, Technion – Israel Institute of Technology. Animal care and research protocols were in accordance with institutional guidelines and were approved by the Institutional Committee on Animal Use (17-08-115-IL).

### Culture of Peripheral blood mononuclear cells

PBMCs were isolated from healthy donors without any history of AA or other autoimmune diseases by centrifugation on Ficoll/Hypaque (Pharmacia, Amersham Pharmacia Biotech, Uppsala, Sweden) (Ghraieb et al., 2018). The PBMCs were then cultured for 14 days with 100 U IL-2 per ml (Pepro Tech Inc, Rocky Hill, NJ) in a medium composed of RPMI 1640, 10% human AB serum (Sigma, St. Louis, MO), 1% glutamine, 1% antibiotics (media components; Biological Industries, Kibbutz Beit Haemeck, Israel). Medium was changed as needed. The cultured cells defined as enriched CD8/NKG2D according to our previous publication (Ghraieb et al., 2018), were injected intradermally into human explants on beige-SCID mice.

### Study design

Two sets of experiments were performed: In the first set, the mice were divided randomly into three groups on day 89 after scalp skin transplantation and treated as described in Supplementary file 2.

The second set of experiments was performed to eliminate the confounding influence of resident human T-cells present in the human scalp skin xenotransplants. To this end, anti-CD3/OKT3 antibodies (Supplementary file 2) were injected into xenotransplants treated with either autologous ILC1lc or autologous enriched CD8+/NKG2D+ cells. For both sets of experiments, the mice were sacrificed and skin biopsies were taken for analysis on day 45 after immunocyte injection.

### Statistical analysis

Data are presented as the mean ± standard error of mean (SEM) or fold change of mean ± SEM; p values of <0.05 were regarded as significant.

Gaussian distribution of the data was analyzed using Shapiro-Wilk test. Significant differences were analyzed using either unpaired Student′s t-test (comparison between one set of data), or One Way ANOVA (comparison between multiple sets of data) for parametric data, or Mann–Whitney test (comparison between one set of data and sham or vehicle) for nonparametric data or Kruskal–Wallis test, and Dunn’s test (comparison between multiple sets of data). The n (e.g. number of donors, tissue sections, or microscopic fields) used for each individual data reported here is listed in corresponding figure legend.

## Data Availability

Source Data files have been provided for Figures 1-8 and figures supplement 2,3,4.

## Additional files

Supplementary file 1. Microdissected, organ-cultured HFs are ‘stressed’ on day 1, but become equilibrated on day 3, as assessed by the listed objective read-out parameters that indicate a temporarily weakened HF immune privilege and HF damage as well as MICA overexpression on day 1. Instead, the expression of HF immune privilege guardians (αMSH, TGFß2) (Bertolini et al., 2020) is preserved. This makes freshly microdissected healthy human scalp HFs one day after initiation of HF organ culture optimally suited as ‘stressed’ human (mini-) organs that strongly express the NKG2D-activating ‘danger’ ‘signal,’ MICA, which also is overexpressed by human AA HFs (Li et al., 2016) (these data are repeated from Uchida et al., 2021 to illustrate the HF distress/partial IP collapse of microdissected human scalp HFs one day 1 after initiation of organ culture).

Supplementary file 2. Route of injections, volume, and the number of immune cells injected into the normal healthy human xenotransplants.

MDAR checklist
