# Supplementary material for: Involvement of ILC1-like innate lymphocytes in human autoimmunity, lessons from alopecia areata
Source: eLife. 2023 Mar 17;12:e80768. doi: 10.7554/eLife.80768 (PMC10023162; doi:10.7554/eLife.80768)
Supplement: Supplementary file 1. — Instead, the expression of HF immune privilege guardians (αMSH, TGFß2) (Bertolini et al., 2020) is preserved. This makes freshly microdissected healthy human scalp HFs one day after initiation of HF organ culture optimally suited as ‘stressed’ human (mini-) organs that strongly express the NKG2D-activating ‘danger’ ‘signal,’ MICA, which also is overexpressed by human AA HFs (Li et al., 2016) (these data are repeated from Uchida et al., 2021 to illustrate the HF distress/partial IP collapse of microdissected human scalp HFs one day 1 after initiation of organ culture). [file elife-80768-supp1.docx]

*** Supplementary file 1- Microdissected, organ-cultured HFs are “stressed” on day 1, but become equilibrated on day 3**

| **Parameter**  **analyzed**  **Hair**  **follicle** | **LDH activity** | **NKG2D ligand MICA** | **CXCL12** | **IP collapse** | | **IP guardians** | |
| --- | --- | --- | --- | --- | --- | --- | --- |
|  |  |  |  | **MHC class I** | **β2-microglobulin** | **α-MSH** | **TGFβ2** |
| **Stressed**  **(1 day in culture)** | Detected | Highly detected | Detected | Detected | Detected | Detected | Highly detected |
| **Non-Stressed**  **(3 days in culture)** | Not detected | Not detected | Not detected | Not detected | Not  detected | Detected | Highly detected |

* Taken from Uchida et al., 2021
