# Supplementary material for: Involvement of ILC1-like innate lymphocytes in human autoimmunity, lessons from alopecia areata
Source: eLife. 2023 Mar 17;12:e80768. doi: 10.7554/eLife.80768 (PMC10023162; doi:10.7554/eLife.80768)
Supplement: Supplementary file 2. [file elife-80768-supp2.docx]

**Supplementary file 2.**

|  | **Treated group** | **Number of Injected Cells** | **Route** | **Injection Volume** | **Number of Mice** |
| --- | --- | --- | --- | --- | --- |
| **Experiment**  **1** | Enriched CD8/NKG2D cells | 4X10^5^ cells | i.d | 100 µl | 3 |
|  | PHA activated PBMCs | 4X10^5^ cells | i.d | 100 µl | 3 |
|  | ILC1lc | 4X10^5^ cells | i.d | 100 µl | 4 |
| **Experiment**  **2** | ILC1lc | 4X10^5^ cells | i.d | 100 µl | 2 |
|  | ILC1lc + anti-CD3 Abs (OKT3, Miltenyi Biotech) | 4X10^5^ cells | i.d | 100 µl | 2 |
|  | Enriched CD8/NKG2D cells | 4X10^5^ cells | i.d | 100 µl | 2 |
|  | Enriched CD8/NKG2D cells + anti-CD3 Abs (OKT3, Miltenyi Biotech) | 4X10^5^ cells | i.d | 100 µl | 2 |
