# Supplementary material for: Involvement of ILC1-like innate lymphocytes in human autoimmunity, lessons from alopecia areata
Source: eLife. 2023 Mar 17;12:e80768. doi: 10.7554/eLife.80768 (PMC10023162; doi:10.7554/eLife.80768)
Supplement: Peer review history

# Editor report: Editor's evaluation

**DOI:** 10.7554/eLife.80768.sa0

**Authors:** Valerie Horsley

**Affiliations:** https://ror.org/03v76x132 Yale University United States

This manuscript provides fundamental data that implicate ILC1-like cells in alopeia areata. The data are solid in the use of cultured human hair follicles co-cultured with ILC-1-like cells and demonstration that alopecia phenotypes emerge. The authors also provide compelling evidence that injection of ILC1-like cells induces alopecia in a mouse model grafted with human hair follicle-containing skin. This work will be of interest to immunologists, skin biologists, and scientists interested in autoimmune disorders.

# Decision letter

**DOI:** 10.7554/eLife.80768.sa1

**Affiliations:** https://ror.org/03v76x132 Yale University United States; https://ror.org/043sz6670 Sinclair Dermatology Australia

In the interests of transparency, eLife publishes the most substantive revision requests and the accompanying author responses.

**Decision letter after peer review:**

Thank you for submitting your article "Involvement of ILC1-like innate lymphocytes in human autoimmunity, lessons from alopecia areata" for consideration by *eLife*. Your article has been reviewed by 3 peer reviewers, and the evaluation has been overseen by a Reviewing Editor and Betty Diamond as the Senior Editor. The following individual involved in the review of your submission has agreed to reveal their identity: Rodney Sinclair (Reviewer #2).

The reviewers have discussed their reviews with one another, and the Reviewing Editor has drafted this to help you prepare a revised submission.

Essential revisions:

1. Clarify the color differences between white and yellow in their triple staining of immunofluorescent photo data in Figure 1A-C, either in the figure legend or the Materials and methods section.

2. Please provide the data for the effect of non-autologous ILC1lc on AA patients ex vivo with co-culture or in vivo with injection are available as references.

3) The reviewers should discuss whether 5% Minoxidil is required for hair regrowth in this system given that the efficacy of Minoxidil has not been convincing to all subjects even for the human species in the previous clinical studies.

4. The authors should update their phenotyping data and subsequent sorting strategy to provide more definitive evidence for the involvement in an ILC1 or tissue-resident NK cell population in AA.

*Reviewer #1 (Recommendations for the authors):*

The authors provide useful data implicating a subset of type 1 innate lymphocytes in the pathogenesis of human AA. Strengths include mini organ models and humanized mouse models, and weaknesses involve inadequate characterization of ILC1-like populations to confidently conclude that the observed cell type is not of the NK cell lineage. This weakness restricts the impact of the manuscript given the author's previous findings on NK cells and AA pathogenesis. The authors need to provide definitive evidence that the reported "ILC1-like" cells are not an activation state of NK cells to support the conclusions and novelty claims written in the manuscript.

– Although previous literature has associated CD49a expression with the ILC1 lineage in mice and humans, CD49a is upregulated on activated NK cells (EOMES+), and EOMEShi TBETlo liver-resident NK cells have been described in humans. Recent single-cell RNAseq studies in mice and human tissues support the conclusion that EOMES+ ILCs represent the NK cell lineage. These findings are directly at odds with the author's main conclusions and are not discussed in the manuscript. The author's phenotype more closely resembles a tissue-resident or activated NK cell rather than an ILC1. Without further evidence of ILC1 lineage-defining markers (CD200R, granzyme C, etc.) or single cell RNAseq validation, the presented phenotyping data is inadequate to support the authors' claims. If evidence is presented to suggest that "ILC1-like cells" closely resemble a tissue-resident NK cell, then there would be no loss of impact on the manuscript.

– It is not clear from Figure S5 whether the authors can conclude that only ILC1-like cells were sorted into this fraction Lin-CD127+NKp44+ cells could still contain ILC3s capable of producing IFNγ. The authors need to formally exclude the possibility that contamination from ILC3s during sorting can contribute to their observed results. This is a caveat of previous studies that the author cites as well.

– ILC1 phenotyping in humans is controversial, however, the addition of more surface markers and single-cell RNAseq data will significantly improve the interpretation of the study. CXCR6, CD200R (ILC1) and Perforin, NKp80, CD16 (NK cells) staining can all be used to further support the author's conclusions. Lack of IRF8 and Eomes expression would signify strong evidence that ILC-like cells are not of the NK cell lineage. ILC1 gene signatures can be sourced from previously published studies in mice and humans to help with unbiased analysis of cell lineages.

– It is suggested that the authors update their phenotyping data and subsequent sorting strategy to provide more definitive evidence for the involvement in an ILC1 or tissue-resident NK cell population in AA.

*Reviewer #2 (Recommendations for the authors):*

Recent major breakthroughs in our understanding of the pathogenesis of AA have led to highly effective FDA-approved treatments for this potentially disfiguring disease. As these treatments require maintenance dosing to prevent relapse, further studies are required to identify the initiating events for hair follicle auto-immunity leading to AA. Natural killer cells expressing cell surface NKG2D transmembrane proteins are already implicated in the pathogenesis of AA. The authors identified and isolated a novel population of NKG2D+ cells – the ILC1-like lymphocytes – that are strategically localized around hair bulbs, that respond to hair follicle distress signals, that play a very early role in the pathogenesis of AA through robust secretion of IFN-γ, leading to loss of hair follicle immune privilege and also a later ole in AA pathogenesis through induction of hair fibre dystrophy and induction of catagen. in vivo studies using mouse xenograft models confirmed that ILCS-a-like cells can induce the AA hair loss phenotype.

This manuscript makes a very important contribution to our understanding of the molecular biology of AA and suggests novel therapeutic targets for future investigation.

A great body of work summarized in a single paper.

*Reviewer #3 (Recommendations for the authors):*

This manuscript intensively studied the direct involvement of ILC1 cells on the onset of AA., cells which seem to be very difficult, characterized by no existence of a single clear marker, and its relatively few populations in the HF even in the lesion of AA patients. To overcome this situation, the authors nicely used a combination of multiple reliable markers such as Enomes, NKG2D, CD49a, and confirmed its location and existence very convincingly.

Authors also carefully define the ILC1 cells as "ILC1-like cells (ILC1-lc), which is also a sincere attitude by the authors on scientific accuracy.

This is excellent work and a comprehensive set of various experiments to confirm the involvement of ILC1-lc on the onset of AA.

Although ex vivo and in vivo models in this manuscript each still have their limitations, such as no systemic system involved or host species difference, nevertheless, they seem to be highly valued as studies using the best model systems currently conceived for AA disease.

In the future, it is expected that clinical data indicating the direct involvement of ILC-1 in AA obtained from the results of this study will be accumulated, and the treatment of current AA patients will be further improved by this finding.

This manuscript is very comprehensive, and all necessary experiments to prove the involvement of ILC1lc for AA disease seem to be performed including both positive and negative controls. It is a very impressive and intensive study for me.

# Reply: Author response

**DOI:** 10.7554/eLife.80768.sa2

Essential revisions:

1. Clarify the color differences between white and yellow in their triple staining of immunofluorescent photo data in Figure 1A-C, either in the figure legend or the Materials and methods section.

Thanks. We have now clarified the color differences between white and yellow in our triple staining of immunofluorescent photo data in Figure 1A-C and Figure 8A-C, as follows:

“For each panel yellow staining indicates double staining (A-EOMES+, NKG2D+; B- EOMES+,CD49a+ ; C- NKp44+,CD103+).”

2. Please provide the data for the effect of non-autologous ILC1lc on AA patients ex vivo with co-culture or in vivo with injection are available as references.

We respectfully propose that this is a questionable and dispensable request. In this study, only the effect of autologous ILC1lc on healthy scalp skin xenotransplants is clinically relevant in the context of alopecia areata (AA), since heterologous ILC1lc cells might induce an (entirely irrelevant and likely misleading) graft-versus-host scenario. This would cause a pathobiologically completely different, scarring type of folliculitis – which is exactly what one must avoid in AA in vivo models (for detailed discussion: see Gilhar et al. Autoimmun Rev 2016).

Using allogeneic ILC1lc would also be physiologically and clinically irrelevant, as this constellation almost never occurs in AA (except in the vanishly rare number of cases of AA after an allogeneic bone marrow transplantation). Moreover, the suggested control experiment could also be criticized for being wasteful, as it violates the widely accepted animal experimentation principle to only use the number of animals that is indispensable for answering the relevant research question(s) at hand.

While we agree that studying this would, nevertheless, be academically interesting, this clearly lies outside of our study scope and would cause major unjustifiable additional costs and loss of time.

3) The reviewers should discuss whether 5% Minoxidil is required for hair regrowth in this system given that the efficacy of Minoxidil has not been convincing to all subjects even for the human species in the previous clinical studies.

Thanks for raising this excellent point. We now explain in the revised Materials and methods section that topical minoxidil application is not required for hair regrowth induction after the initial post-transplantation hair shaft shedding, but only accelerates it, and that this application is discontinued before the ILC1lc injection (Gilhar et al. JID 2013). Since the immune cell infiltrate in AA attacks only hair follicles in anagen (Gilhar et al. NEJM 2012), it is critical that the majority of xenotransplanted HFs are in anagen at the time the immune cells are injected. Topical minoxidil pretreatment increases the likelihood that this is the case (Price, NEJM 1999; Suchonwanit et al. Drug Des Devel Ther 2019).

Moreover, we have recently demonstrated that 5% minoxidil does indeed significantly stimulate hair regrowth in human androgenetic alopecia scalp skin transplanted onto SCID/beige mice (Gilhar et al. Exp Dermatol 2022). It also deserves mentioning that topical minoxdil reduces the degranulation of – hair growth-modulatory! (Paus et al. Dev Biol 1994) – perifollicular mast cells in the skin of mice, namely under conditions of perceived stress (Arck et al. Exp Dermatol 2003), while excessive degranulation of perifollicular mast cells is an important feature of lesional human AA skin (Bertolini et al. PLoS One 2014). Therefore, this pretreatment likely also helps to reestablish perifollicular mast cell homeostasis after the stress of xenotransplantation.

These explanations are now given on pages 34 and 35:

“The topical minoxidil application is not required for hair regrowth induction after the initial post-transplantation hair shaft shedding, but only accelerates it. This application is discontinued before the ILC1lc injection (Gilhar et al., 2013).Since the immune cell infiltrate in AA attacks only hair follicles in anagen (Gilhar et al., 2012), it is critical that the majority of xenotransplant HFs are in anagen at the time the immune cells are injected. Topical minoxidil pretreatment increases the likelihood that this is the case (Price, 1999; Suchonwanit et al., 2019). Moreover, we have recently demonstrated that 5% minoxidil does indeed significantly stimulate hair regrowth in human androgenetic alopecia scalp skin transplanted onto SCID/beige mice (Gilhar et al., 2022). It also deserves mentioning that topical minoxdil reduces the degranulation of – hair growth-modulatory! (Paus et al., 1994) – perifollicular mast cells in the skin of mice, namely under conditions of perceived stress (Arck et al., 2003), while excessive degranulation of perifollicular mast cells is an important feature of lesional human AA skin (Bertolini et al., 2014). Therefore, this pretreatment likely also helps to reestablish perifollicular mast cell homeostasis after the stress of xenotransplantation.”

4. The authors should update their phenotyping data and subsequent sorting strategy to provide more definitive evidence for the involvement in an ILC1 or tissue-resident NK cell population in AA.

Thank you for this important suggestion. In order to provide more definitive evidence for the involvement in an ILC1 or tissue-resident NK cell population in AA, we now have re-analyzed the sorted ILC1lc by FACS analysis, using the additional ILC1lc markers CXCR6, CD200R (Curio et al. Cell Mol Immunol 2022) and the classical NK markers IRF8, perforin, NKp80, and CD16 (Brownlie et al. PNAS 2021; Krämer et al. Cell Rep 2023; Gilhar et al. JID 2013). Granzyme C is expressed by murine and not human NK cells and therefore has not been used in the current study (Susanto et al. Tissue Antigens 2012; Cai et al. J Immunol 2009). This shows more definitive evidence for the involvement of ILC1 cell population in AA. These novel data are now reported in the results (page 10) and in a new Figure 2—figure supplement 1.

Reviewer #1 (Recommendations for the authors):

The authors provide useful data implicating a subset of type 1 innate lymphocytes in the pathogenesis of human AA. Strengths include mini organ models and humanized mouse models, and weaknesses involve inadequate characterization of ILC1-like populations to confidently conclude that the observed cell type is not of the NK cell lineage. This weakness restricts the impact of the manuscript given the author's previous findings on NK cells and AA pathogenesis. The authors need to provide definitive evidence that the reported "ILC1-like" cells are not an activation state of NK cells to support the conclusions and novelty claims written in the manuscript.

We fully agree with this expert reviewer in principle, but must reemphasize how challenging it is to “definitively” distinguish between ILC1 and NK cells (even all experts on this do not seem to be able to fully agree on how to best do so…).

In fact, how to reliably discriminate between NK cells and ILC1s and unravelling the shared and distinct functions of these cell populations remains an important open quest (Seillet et al. Curr Opin Immunol 2021; Lopes et al. Semin Immunol 2023; Cheng et al. Cell Mol Gastroenterol Hepatol 2023; Taggenbrock et al. Eur J Immunol 2022). Therefore, distinction between NK cells and ILC1s remains provisional – which is exactly why we have cautiously labeled the latter as “ILC1lc”.

We have now further underscored this in the revised Introduction, pages 5 and 6.

"In fact, how to reliably discriminate between NK cells and ILC1s and unravelling the shared and distinct functions of these cell populations remains an important open quest (Seillet et al., 2021; Lopes et al., 2023; Cheng et al., 2023; Taggenbrock and van Gisbergen, 2022). For example, Eomes^hi^ T-bet^lo^ liver-resident NK cells have been described in humans and mice (Park et al., 2019; Harmon et al., 2016), while ILC1s from human tonsil and blood were also found to be Eomes+ (Cella et al., 2019). Therefore, distinction between NK cells and ILC1s remains provisional – which is exactly why we have cautiously labeled the latter as “ILC1lc."

In order to provide even more persuasive evidence than before that the reported "ILC1-like" cells (ILC1lc) are not just activated NK cells, and to support the conclusions and novelty claims contained in our study, we have performed additional FACS analyses with multiple established key markers of NK versus ILC1lc (details: see above). These new data clearly support our claims (new Figure 2—figure supplement 1 A-C).

– Although previous literature has associated CD49a expression with the ILC1 lineage in mice and humans, CD49a is upregulated on activated NK cells

Thank you for pointing this out. An upregulation of CD49a on activated human and murine NK cells has indeed been reported (Albini et al. Cancers (Basel) 2021; Zheng et al. J Immunol 2016). To correctly reflect this, we have reworded the corresponding text in the revised Introduction (page 6):

“However, although integrin α1 (CD49a) is upregulated on activated NK cells (Albini et al., 2021; Zheng et al., 2016), CD49a and integrin α2 (CD49b) are used as two mutually exclusive markers for distinguishing between NK and ILC1 cells, with NK cells being defined as CD49b+CD49a- and ILC1 as CD49b-CD49a+ (Gao et al., 2017; Vienne et al., 2021; Flommersfeld et al., 2021; Krzywinska et al., 2022) In the current study, we have accepted and employed this consensus."

(EOMES+), and EOMEShi TBETlo liver-resident NK cells have been described in humans.

Thanks again for raising this important point. Indeed, Eomes^hi^ T-bet^lo^ liver-resident NK cells have been described in humans and mice (Park et al. *eLife* 2019; Harmon et al. Eur J Immunol 2016). However ILC1s from human tonsil and blood were also found to be Eomes+ (Cella et al. Nat Immunol 2019; Mazzurana et al. Cell Res 2021), further indicating the importance to compare the expression levels of additional key NK and ILC1 cell markers on both cell populations (see introduction, pages 5 and 6 and above). This has now been done by the new FACS analysis we have integrated into the R.1 manuscript (new Figure 2—figure supplement 1 A-C and Results section, page 10):

"This immune phenotype suggests that the immune cells used in our study are best classified as ILC1lc (Nabekura et al., 2021a), and documents that all experiments reported below were indeed performed with autologous ILC1lc rather than with NK cell subpopulations. Indeed, the FACS analysis (Figure 2 – figure supplement 1A,B and C) revealed that ILC1lc demonstrate the ILC1 phenotype (CD200R, CD127, CXCR6) (Lopes et al., 2023; Curio and Belz, 2022) but not of the classical NK cell lineage (IRF8, Perforin, NKp80, CD16) (Brownlie et al., 2021; Krämer et al., 2023; Sagebiel et al., 2019), thus further serving as an evidence that EOMES+ ILCs represent distinct ILC1 lineage-defining markers"

Recent single-cell RNAseq studies in mice and human tissues support the conclusion that EOMES+ ILCs represent the NK cell lineage. These findings are directly at odds with the author's main conclusions and are not discussed in the manuscript.

Once again, the reviewer raises an excellent point. However, the ILC1lc analyzed in our study are highly unlikely to represent the NK cell lineage because our new data demonstrate that ILC1lc are positive for, CD200R, CD127, CXCR6 but negative for IRF8, Perforin, NKp80, CD16 (new Figure 2—figure supplement 1 A-C). We could not use granzyme C as NK marker, since it is expressed only in mice and not in human (Susanto et al. Tissue Antigens 2012; Cai et al. J Immunol 2009).

The author's phenotype more closely resembles a tissue-resident or activated NK cell rather than an ILC1. Without further evidence of ILC1 lineage-defining markers (CD200R, granzyme C, etc.) or single cell RNAseq validation, the presented phenotyping data is inadequate to support the authors' claims. If evidence is presented to suggest that "ILC1-like cells" closely resemble a tissue-resident NK cell, then there would be no loss of impact on the manuscript.

For the reasons delineated above, we respectfully disagree with this expert referee’s verdict. The following additional data and arguments further support our claim that the ILC1lc analyzed here indeed have much more in common ILC1 than NK characteristics:

1. EOMES expression by ILCs was observed differently dependent on tissue localization (McFarland et al. Immunity 2021).

2. Recent single-cell RNA-sequencing study reveals that ILC1lc both in blood and tonsil are Eomes positive (Mazzurana et al. Cell Res 2021).

3. Other study demonstrated that intraepithelial ILC1s from human tonsil were found to be heterogeneous, encompassing Eomes− and Eomes+ subsets (Cella, et al. Nat Immunol 2019).

Therefore, it is our carefully considered opinion that it would be unreasonable to demand additional data in support of the ILC1-like nature of “our” cells. However, in the revised Discussion, we have now openly acknowledged and cautiously highlighted that “…This claim remains limited by the set of biomarkers that is currently available (and can reasonably be expected to be applied) to “definitively” distinguish between ILC1 and NK lineages, namely in human skin, and that our data may eventually have to be re-evaluated in the context of research progress in this fast-moving field.” (page 19).

This is now stated clearly in the revised manuscript as follows in the M and M (page 32):

“Cells were incubated for 25 minutes at room temperature in the dark. All tubes were washed once with 1 ml staining buffer, then Fixation/Permeabilization solution (250 μl) was added and cells were incubated for 20 minutes at 4^o^C. Cell permeability was performed using 1xBD Perm/Wash buffer, intra-cellular antibody mixtures 50 μl/Brilliant Violet 605TM anti-T-bet (BLG 644817), Eomes-conjugated-PerCP-eFluor 710 (Dan11mag), IRF8 (sc-365042), Perforin (BLG-308119) and INF-γ-conjugated- PE/VIO-770 (Miltenyi Biotec 130-109-313), CD49a-APC-Vio770 (Miltenyi Biotec 130-101-324) ,FITC anti-human CD49b (BLG 359305), APC anti-human NKp80 (BLG 346707), BV421 anti-human CXCR6 (BLG 356013) and APC/Fire 810 anti-human CD16 (BLG 302073) and BV421 anti-human RORγt were added and incubated for 30 minutes at room temperature in the dark, cells were then washed twice with 1xBD Perm/Wash buffer (BD Cytofix/CytopermTM Fixation/Permeabilization Kit).

All cell samples were detected by FACS Calibur Flow Cytometer (Benton Dickinson) using Cell Quest software, and the acquired data were further analyzed using FlowJo 5.7.2 (Tree Star).”

In the abstract

“This provides the first evidence that ILC1lc, which are positive for the ILC1 phenotype and negative for the classical NK markers, suffice to induce AA in previously healthy human HFs ex vivo and in vivo, and further questions the conventional wisdom that AA is always an autoantigen-dependent, CD8^+^ T cell-driven autoimmune disease."

Results (see above and page 10).

Discussion (pages 18 and 19):

"The current study is the first to phenotypically and functionally explore the role of Eomes+ ILC1lc in human AA in vivo and ex vivo. Eomes+ ILCs may represent the NK cell lineage and therefore the ILC1 phenotype in our study, more closely resembles a tissue-resident or activated NK cell rather than an ILC1, based on recent single-cell RNAseq studies in mice and human tissues (Lopes et al., 2023). However, Eomes expression by ILCs was observed differently depending on the tissue localization (McFarland et al., 2021). For example, recent single-cell RNA-sequencing study reveals that ILC1lc both in blood and tonsil are Eomes positive (Mazzurana et al., 2021). Other study demonstrated that intraepithelial ILC1s from human tonsil were found to be heterogeneous, encompassing Eomes− and Eomes+ subsets (Cella et al., 2019). It is indeed very important to obtain greater clarity on how ILC1lc and NKs differ from each other, namely in the context of human AA. Our new data, which demonstrate that ILC1lc are positive for CD49a, CD200R, CD127, CXCR6 (Lopes et al., 2023) but negative for IRF8, Perforin, NKp80, CD16 (Sagebiel et al., 2019) (Figure 2 – figure supplement 1A,B and C), strongly support that ILC1lc and NK cells have distinct lineages. This claim remains limited by the set of biomarkers that is currently available (and can reasonably be expected to be applied) to “definitively” distinguish between ILC1 and NK lineages, namely in human skin, and that our data may eventually have to be re-evaluated in the context of research progress in this fast-moving field. It would be desirable to further complement these results in future studies with unbiased scRNAseq data, which can then be compared with published human ILC1 and NK cell gene signatures so as to gain deeper insights into the – still controversial and unclear – transcriptional similarities and differences between ILC1lc and NK cells and their lineage relationship to each other."

– It is not clear from Figure S5 whether the authors can conclude that only ILC1-like cells were sorted into this fraction Lin-CD127+NKp44+ cells could still contain ILC3s capable of producing IFNγ. The authors need to formally exclude the possibility that contamination from ILC3s during sorting can contribute to their observed results. This is a caveat of previous studies that the author cites as well.

We fully agree with the reviewer's comment and therefore have now formally excluded the possibility that contamination from ILC3s during sorting may have contributed to our results by generating a new set of FACS data on sorted ILC1lc. Given that ILC1 cells are RORγt negative while ILC3 cells are RORγt positive (Peng et al. PNAS 2022; Fiancette et al. Nat Immunol 2021), these novel data render the contamination hypothesis highly unlikely (see Figure 2—figure supplement 1 D). This is now stated clearly in the revised Materials and methods and Results sections.

Materials and methods (page 32)

“Cells were incubated for 25 minutes at room temperature in the dark. All tubes were washed once with 1 ml staining buffer, then Fixation/Permeabilization solution (250 μl) was added and cells were incubated for 20 minutes at 4^o^C. Cell permeability was performed using 1xBD Perm/Wash buffer, intra-cellular antibody mixtures (50 μl/Brilliant Violet 605TM anti-T-bet (BLG 644817)), Eomes-conjugated-PerCP-eFluor 710 (Dan11mag), IRF8 (sc-365042), Perforin (BLG-308119) and INF-γ-conjugated- PE/VIO-770 (Miltenyi Biotec 130-109-313), CD49a-APC-Vio770 (Miltenyi Biotec 130-101-324) ,FITC anti-human CD49b (BLG 359305), APC anti-human NKp80 (BLG 346707), BV421 anti-human CXCR6 (BLG 356013) and APC/Fire 810 anti-human CD16 (BLG 302073) and BV421 anti-human RORγt were added and incubated for 30 minutes at room temperature in the dark, cells were then washed twice with 1xBD Perm/Wash buffer (BD Cytofix/CytopermTM Fixation/Permeabilization Kit)..

All cell samples were detected by FACS Calibur Flow Cytometer (Benton Dickinson) using Cell Quest software, and the acquired data were further analyzed using FlowJo 5.7.2 (Tree Star).”

Result section (page 11):

“In order to exclude the possibility that contamination from ILC3s during sorting the ILC1lc and thus contributing to the observed results, we generated a new set of FACS data on sorted ILC1lc. Given that ILC1 cells are RORγt negative while ILC3 cells are RORγt positive (Peng et al., 2022; Fiancette et al., 2021), the data clearly demonstrate that the contamination hypothesis is highly unlikely (Figure 2 – figure supplement 1D).”

– ILC1 phenotyping in humans is controversial, however, the addition of more surface markers and single-cell RNAseq data will significantly improve the interpretation of the study. CXCR6, CD200R (ILC1) and Perforin, NKp80, CD16 (NK cells) staining can all be used to further support the author's conclusions. Lack of IRF8 and Eomes expression would signify strong evidence that ILC-like cells are not of the NK cell lineage. ILC1 gene signatures can be sourced from previously published studies in mice and humans to help with unbiased analysis of cell lineages.

Again, we fully agree with the reviewer's comment and have therefore engaged in more rigorous ILC1 phenotyping, including by investigating whether “our” ILC1lc lack IRF8 and Eomes surface marker expression. Our new data show that this is indeed the case (see new Figure 2—figure supplement 1 A-C), thus further supporting our previous claim that ILC-like cells are not of the NK cell lineage. This is now stated clearly in the revised Abstract and Results (page 10) and is explained in the revised Discussion (pages 18 and 19).

We also accept that single-cell RNAseq analysis and comparison with published data may help with unbiased analysis of cell lineages. However, this time-consuming and costly analysis far exceeds our available budget for this study and, in view of new, more relevant data (new Figure 2—figure supplement 1 A-C), also appears dispensable to support our claims. Moreover the costs for the ILC1 antibodies are extremely high and we simply do not have the funds for this, and that we therefore had to go for the FACS approach.

Yet, we now explicitly acknowledge in the revised Discussion (Page 19) that:

“It would be desirable to further complement these results in future studies with unbiased scRNAseq data, which can then be compared with published human ILC1 and NK cell gene signatures so as to gain deeper insights into the – still controversial and unclear – transcriptional similarities and differences between ILC1lc and NK cells and their lineage relationship to each other.”

– It is suggested that the authors update their phenotyping data and subsequent sorting strategy to provide more definitive evidence for the involvement in an ILC1 or tissue-resident NK cell population in AA.

Thanks again so much for these very helpful, important, and constructive suggestions!

Reviewer #2 (Recommendations for the authors):

Recent major breakthroughs in our understanding of the pathogenesis of AA have led to highly effective FDA-approved treatments for this potentially disfiguring disease. As these treatments require maintenance dosing to prevent relapse, further studies are required to identify the initiating events for hair follicle auto-immunity leading to AA. Natural killer cells expressing cell surface NKG2D transmembrane proteins are already implicated in the pathogenesis of AA. The authors identified and isolated a novel population of NKG2D+ cells – the ILC1-like lymphocytes – that are strategically localized around hair bulbs, that respond to hair follicle distress signals, that play a very early role in the pathogenesis of AA through robust secretion of IFN-γ, leading to loss of hair follicle immune privilege and also a later ole in AA pathogenesis through induction of hair fibre dystrophy and induction of catagen. in vivo studies using mouse xenograft models confirmed that ILCS-a-like cells can induce the AA hair loss phenotype.

This manuscript makes a very important contribution to our understanding of the molecular biology of AA and suggests novel therapeutic targets for future investigation.

A great body of work summarized in a single paper.

Thank you kindly for this most encouraging verdict.

Reviewer #3 (Recommendations for the authors):

This manuscript intensively studied the direct involvement of ILC1 cells on the onset of AA., cells which seem to be very difficult, characterized by no existence of a single clear marker, and its relatively few populations in the HF even in the lesion of AA patients. To overcome this situation, the authors nicely used a combination of multiple reliable markers such as Enomes, NKG2D, CD49a, and confirmed its location and existence very convincingly.

Authors also carefully define the ILC1 cells as "ILC1-like cells (ILC1-lc), which is also a sincere attitude by the authors on scientific accuracy.

This is excellent work and a comprehensive set of various experiments to confirm the involvement of ILC1-lc on the onset of AA.

We are most grateful for this assessment.
